# Interaction of the bacterial division regulator MinE with lipid bicelles studied by NMR spectroscopy

**DOI:** 10.1016/j.jbc.2023.103037

**Published:** 2023-02-17

**Authors:** Mengli Cai, Vitali Tugarinov, Sai Chaitanya Chiliveri, Ying Huang, Charles D. Schwieters, Kyoshi Mizuuchi, G. Marius Clore

**Affiliations:** 1 Laboratory of Chemical Physics, National Institute of Diabetes and Digestive and Kidney Diseases, National Institutes of Health, Bethesda, Maryland, USA; 2 Laboratory of Molecular Biology, National Institute of Diabetes and Digestive and Kidney Diseases, National Institutes of Health, Bethesda, Maryland, USA; 3 Computational Biomolecular Magnetic Resonance Core, National Institute of Diabetes and Digestive and Kidney Diseases, National Institutes of Health, Bethesda, Maryland, USA

**Keywords:** MinE, lipid bicelles, residual dipolar couplings, ^15^N relaxation, relaxation dispersion, dynamics, excited states, 16-DSA, 16-doxyl stearic acid, CPMG, 15N Carr-Purcell-Meiboom-Gill, DHPC, 6:0 1,2-dihexanoyl-sn-glycero-3-phosphocholine, DMPC, 14:0 1,2-dimyristoyl-sn-glycero-3-phosphocholine, ngMinE, Neisseria gonorrhoeae MinE, NOE, nuclear overhauser enhancement, PRE, paramagnetic relaxation enhancement, RDC, residual dipolar coupling

## Abstract

The bacterial MinE and MinD division regulatory proteins form a standing wave enabling MinC, which binds MinD, to inhibit FtsZ polymerization everywhere except at the midcell, thereby assuring correct positioning of the cytokinetic septum and even distribution of contents to daughter cells. The MinE dimer undergoes major structural rearrangements between a resting six-stranded state present in the cytoplasm, a membrane-bound state, and a four-stranded active state bound to MinD on the membrane, but it is unclear which MinE motifs interact with the membrane in these different states. Using NMR, we probe the structure and global dynamics of MinE bound to disc-shaped lipid bicelles. In the bicelle-bound state, helix α1 no longer sits on top of the six-stranded β-sheet, losing any contact with the protein core, but interacts directly with the bicelle surface; the structure of the protein core remains unperturbed and also interacts with the bicelle surface *via* helix α2. Binding may involve a previously identified excited state of free MinE in which helix α1 is disordered, thereby allowing it to target the membrane surface. Helix α1 and the protein core undergo nanosecond rigid body motions of differing amplitudes in the plane of the bicelle surface. Global dynamics on the sub-millisecond time scale between a ground state and a sparsely populated excited state are also observed and may represent a very early intermediate on the transition path between the resting six-stranded and active four-stranded conformations. In summary, our results provide insights into MinE structural rearrangements important during bacterial cell division.

A network of three bacterial proteins, MinC, MinD, and MinE, play a key role in ensuring that the position of the cytokinesis-initiating FtsZ polymerization and hence the site of cell division occurs at the midpoint of the cell, thereby ensuring even partitioning of replicated copies of the chromosome and cell contents between daughter cells (1, 2, 3, 4, 5). In the presence of MinE which regulates the interaction of MinD with the membrane and its ATPase activity, MinD and MinE self-organize to form a standing oscillatory wave with a node at the midcell, while MinC, an inhibitor of FtsZ polymerization tracks MinD, such that FtsZ polymerization only occurs at the midcell (6, 7, 8, 9, 10, 11, 12).

During the course of the interaction cycle involving MinD and the cell membrane, the MinE dimer transitions between three distinct conformations: the resting state, present in the cytosol, comprises a six-stranded antiparallel β-sheet sandwiched between two pairs of helices that lie above and below the sheet (Fig. 1*A*, left panel) (13, 14); the dimer core structure of an active state, represented by the Δ30 and Δ10/I24 N deletion constructs (Fig. 1*A*, right panel), and seen in the complex with MinD, in which the central β1 strands are extruded to interact with MinD, leaving behind a four-stranded β-sheet (14, 15, 16); and a putative intermediate state in which the α1 helix, possibly acting as a fly-cast (17), anchors MinE on the membrane prior to interaction with membrane-bound MinD. The state of the central β-sheet in this intermediate membrane-bound structure is unknown, although it has been suggested from circular dichroism that membrane binding is sufficient to convert MinE from the six-stranded to four-stranded conformation (18); also unknown is whether regions of MinE, in addition to helix α1, interact directly with the membrane surface. Here we examine the interaction of *Neisseria gonorrhoeae* MinE (ngMinE) with lipid bicelles by NMR spectroscopy, using a combination of backbone chemical shifts, residual dipolar couplings in an aligned medium, paramagnetic relaxation enhancement (PRE), ^15^N-relaxation studies, and ^15^N relaxation dispersion measurements. We show that the core structure of the ngMinE dimer, comprising the six-stranded β-sheet and two α2 helices, is unperturbed upon its binding to lipid bicelles; the two α1 helices, whose secondary structure is preserved, are no longer in contact with the six-stranded β-sheet but interact directly with the surface of the bicelles; the ngMinE core also binds directly to the bicelle surface *via* the α2 helices; the ngMinE core and α1 helices undergo different amplitude rigid body motions on the surface of the bicelles on the nanosecond timescale; and finally ngMinE interconverts between the major observable species and a sparsely populated excited state that may possibly represent a very early intermediate along the transition path between the six- and four-stranded conformations.

**Figure 1:**
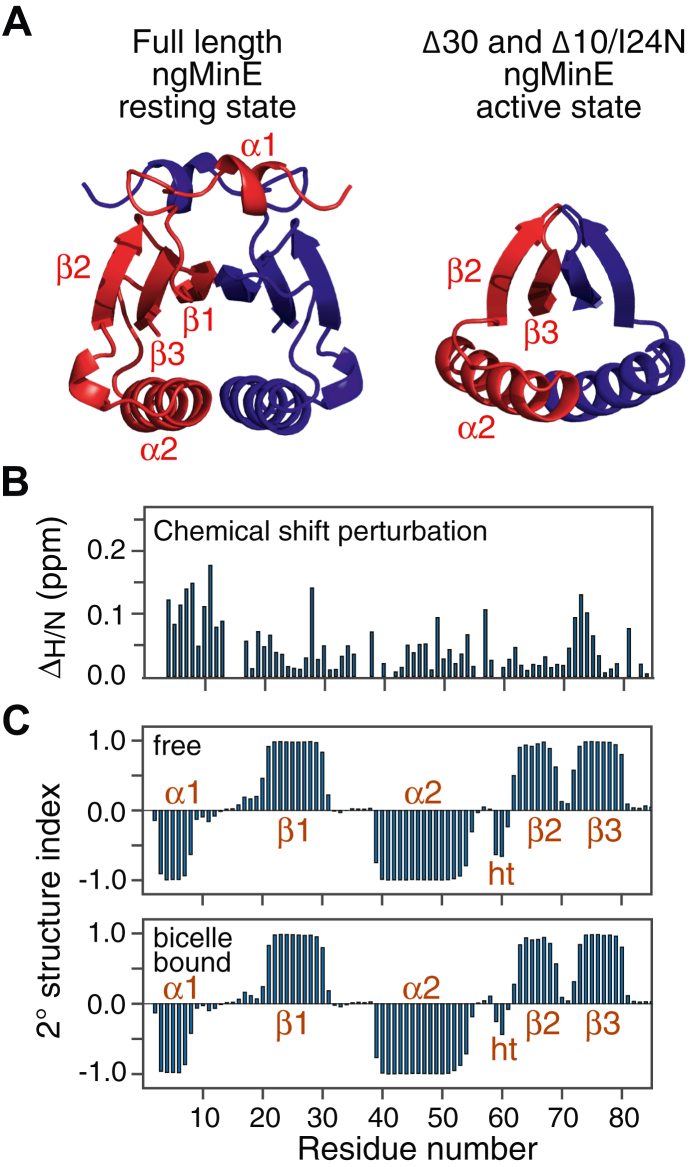
**Interaction of ngMinE with *q* = 0.5 DMPC:DHPC bicelles.***A*, ribbon diagrams of the structures of full-length ngMinE in the resting, six-stranded state (PDB 6U6P), and truncated ngMinE (Δ30 and Δ10/I24N) in the active, four-stranded, state (PDB 6U6R and 6U6S) (14). *B*, ^1^H_N_/^15^N chemical shift difference between free and bicelle-bound full-length ngMinE. Δ_H/N_ (in ppm) is calculated as Δδ = [ Δδ^2^_Η_ + Δδ^2^_Ν_/25]^1/2^ (48), where Δδ_Η_ and Δδ_Ν_ are the backbone ^1^H_N_ and ^15^N chemical shift differences, respectively, between the free and bicelle-bound states. *C*, TALOS secondary structure index for free (*top*) and bicelle-bound (*bottom*) full-length ngMinE derived from backbone ^15^N, ^13^C, and ^1^H chemical shifts (20) (see Tables S1 and S2) Ht, helical turn. DHPC, 6:0 1,2-dihexanoyl-sn-glycero-3-phosphocholine; DMPC, 14:0 1,2-dimyristoyl-sn-glycero-3-phosphocholine; ngMinE, *Neisseria gonorrhoeae* MinE.

## Results and discussion

### Backbone chemical shifts, secondary and tertiary structure of bicelle-bound ngMinE

The interaction of ngMinE with *q* = 0.5 DMPC (14:0 1,2-dimyristoyl-sn-glycero-3-phosphocholine):DHPC (6:0 1,2-dihexanoyl-sn-glycero-3-phosphocholine) bicelles was probed by three-dimensional triple resonance NMR spectroscopy. *q* = 0.5 DMPC:DHPC bicelles form an oblate disc with a DHPC rim; the approximate diameter and thickness of the disc are ∼60 to 70 and 50 Å, respectively, with a molecular weight of ∼ 50 kDa (19). A comparison of the ^1^H-^15^N correlation spectra of free and bicelle-bound ^2^H/^15^N-labeled ngMinE is provided in Fig. S1, and a plot of the combined ^1^H_N_/^15^N chemical shift perturbation (Δ_H/N_) profile is shown in Figure 1*B*. Under the conditions of these experiments, all ngMinE is bicelle-bound, as no evidence of ^1^H-^15^N cross-peaks arising from free ngMinE are observed in the presence of bicelles (Fig. S2). While Δ_H/N_ chemical shift perturbations upon binding the *q* = 0.5 bicelles are seen throughout the ngMinE sequence, the largest perturbations occur in the region of the α1 helix. The TALOS-derived secondary structure index profiles for free and bicelle-bound ngMinE, calculated from backbone (^1^H, ^15^N, ^13^C) shifts (Tables S1 and S2, respectively) (20), indicate that the secondary structure elements, comprising not only the protein core (strands β1-β3 and helix α2) but also helix α1, are preserved upon interaction with the lipid bicelles (Fig. 1*C*). The register of six-stranded β-sheet in the bicelle-bound state is identical to that of the free state, as confirmed by the characteristic pattern of interstrand (both intrasubunit and intersubunit) nuclear overhauser enhancements (NOEs) observed between backbone amide protons in a 3D ^15^N-separated NOE spectrum (Fig. S3).

To further ascertain whether there are any changes in three-dimensional structure of the ngMinE core upon binding bicelles, we measured backbone amide (^1^*D*_NH_) residual dipolar couplings (RDCs) in an aligned medium comprising a stretched, positively charged, polyacrylamide gel (Fig. S4 and Table S3). In an aligned medium, backbone amide RDCs are highly sensitive to the orientation of N-H bond vectors to the principal axis of the external alignment tensor and thus provide a very sensitive probe of three-dimensional structure (21). For residues within the secondary structure elements of the protein core (whose structure is well-defined), there is excellent agreement (Fig. S4) between the experimental RDCs for bicelle-bound ngMinE and those calculated from the coordinates of free ngMinE (PDB 6U6P) (14), previously determined using CS-ROSETTA (22) based on backbone chemical shifts and RDC measurements. The RDC *R*-factor (23) for the protein core is 16%; this value is increased to 23% when four residues from helix α1 are included (Fig. S4), which is consistent with the α1 helix no longer contacting the 6-stranded β-sheet but binding directly to the surface of the lipid bicelles (see next section).

### Sites of interaction of ngMinE with the surface of lipid bicelles probed by PRE

**Figure 2:**
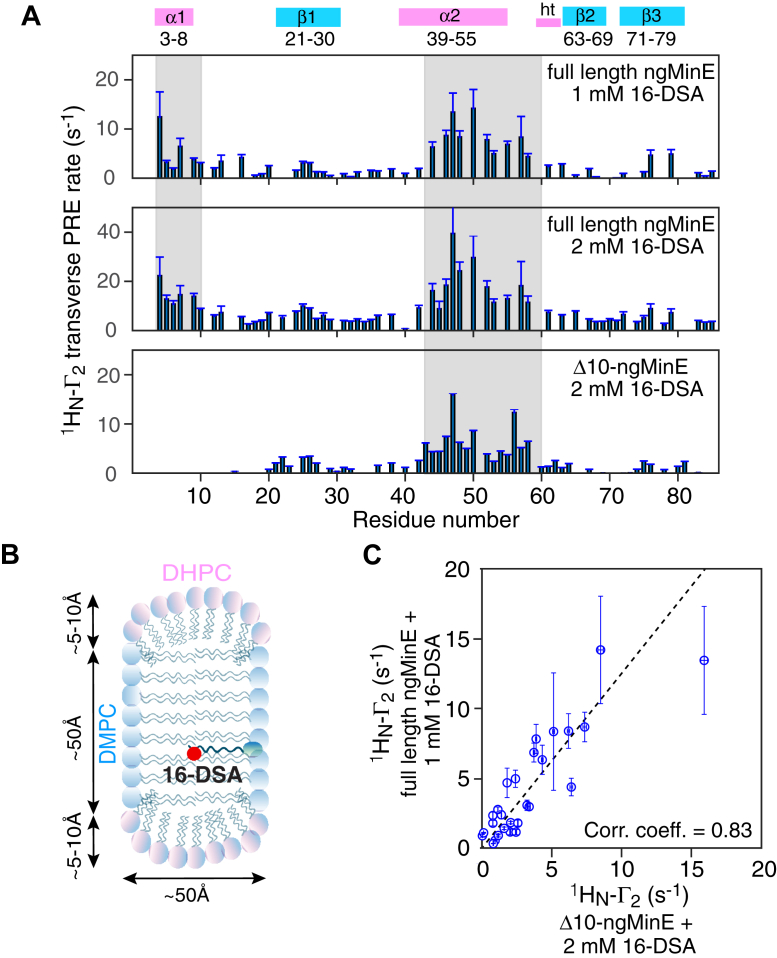
**PRE mapping of the interaction surface for the binding of full-length and Δ10 ngMinE to *q* = 0.5 DMPC:DHPC bicelles.***A*, ^1^H_N_-Γ_2_ transverse PRE profiles measured for 0.5 mM ^15^N/^2^H-labeled ngMinE (full-length and the Δ10 deletion construct), upon binding to 100 mM *q* = 0.5 DMPC:DHPC (33.3 mM DMPC, 66.67 mM DHPC) bicelles dopped with paramagnetic 16-doxyl stearic acid (16-DSA) at the concentrations indicated. *B*, diagrammatic cross-section with approximate dimensions of a *q* = 0.5 DMPC:DHPC bicelle oblate disc (19) with a 16-DSA molecule shown embedded between the lipid aliphatic chains of DMPC and the site of the nitroxide indicated by the *red circle* (24, 25). ngMinE binds to the DMPC moiety and DHPC forms the rim of the bicelles. *C*, correlation between ^1^H_N_-Γ_2_ values measured for full-length and Δ10 bicelle-bound ngMinE. The ^1^H_N_-Γ_2_ values are given by the difference in ^1^H_N_-*R*_2_ values measured in the presence and absence of the 16-DSA nitroxide dopant. The experimental data were recorded at 800 MHz at 35 °C. Error bars: 1 S.D. DHPC, 6:0 1,2-dihexanoyl-sn-glycero-3-phosphocholine; DMPC, 14:0 1,2-dimyristoyl-sn-glycero-3-phosphocholine; ngMinE, *Neisseria gonorrhoeae* MinE; PRE, paramagnetic relaxation enhancement.

To delineate the regions of ngMinE that interact with the surface of lipid bicelles, we carried out PRE measurements in which the *q* = 0.5 DMPC:DHPC bicelles were doped with the nitroxide 16-doxyl stearic acid (16-DSA) (24, 25) (Fig. 2). The ^1^H_N_-Γ_2_ PRE, measured as the difference in ^1^H_N_ transverse relaxation rates in the presence and absence of 16-DSA, provides a measure of the <*r*^−6^> separation between the unpaired electron on 16-DSA and backbone amide protons of ngMinE (26). The ^1^H_N_-Γ_2_ PRE profiles for full-length ngMinE in the presence of 1 and 2 mM 16-DSA are shown in the top and middle panels of Figure 2*A*. As expected, the magnitude of the PREs is linearly proportional to the concentration of 16-DSA, and two interaction surfaces (indicated by the gray bars) are clearly delineated comprising helices α1 and α2, as well as the loop connecting helix α2 to the helical turn that precedes strand β2 (see Fig. 1*A*, left panel). Also shown in Figure 2*A* (bottom panel) is the ^1^H_N_-Γ_2_ PRE profile for the Δ10-ngMinE construct that lacks helix α1. The Δ10-ngMinE construct also interacts weakly (in fast exchange) with the *q* = 0.5 bicelles *via* helix α2 (and the following loop), and although the PREs are approximately half the magnitude of those obtained with full-length ngMinE at the same DSA concentration, owing to reduced occupancy of the bound state of the Δ10-ngMinE construct under the experimental conditions employed, the PREs for the Δ10 and full-length ngMinE constructs are highly correlated (Fig. 2*C*). The PRE data thus establish unambiguously that both helices α1 and α2 interact simultaneously with the bicelle surface and that therefore helices α1 and α2 lie in the same membrane plane in the bicelle-bound state.

### ^15^N-relaxation studies on bicelle-bound ngMinE

**Figure 3:**
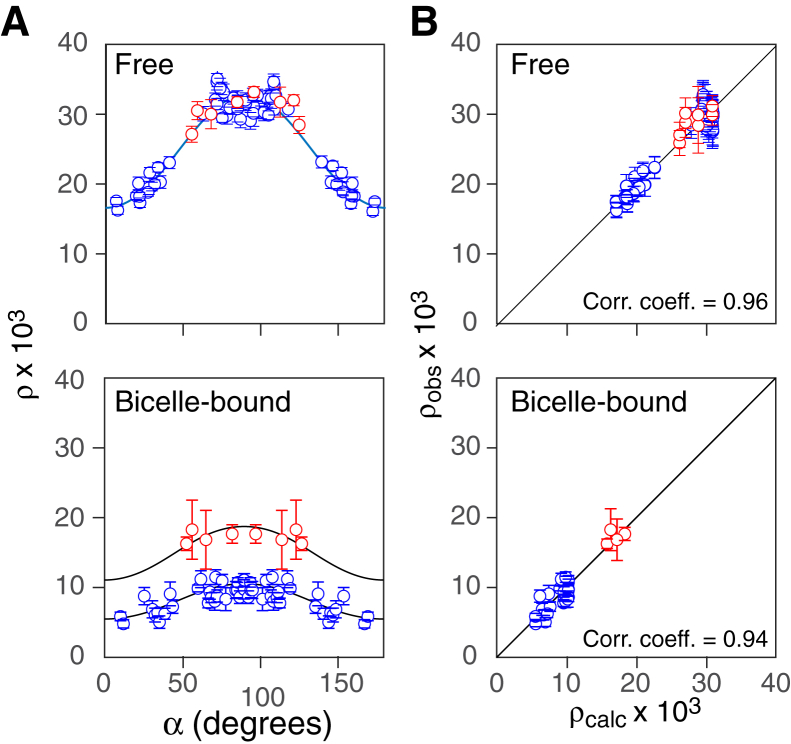
**Analysis of**^**15**^**N-relaxation data for free and bicelle-bound ngMinE.***A*, plot of ρ = *R*_1_/(2*R*_2_ − *R*_1_) *versus* the angle α that the N-H bond vectors make to the principal axis of the diffusion tensor. The *circles* are the experimental data with the α1 helix residues in *red* and the remainder in *blue*, and the *solid lines* represent the best-fit curves with the diffusion parameters given in Table 1. The coordinates used in the best-fitting are those of the NMR structure of free ngMinE (PDB 6U6P; (14)). *B*, correlation plots between observed and calculated ρ values. Equivalent plots for ^15^N-*R*_1_, *R*_2_ and *R*_2_/*R*_1_ are provided in the Fig. S6. The experimental data were recorded on 0.5 mM (in subunits) ^2^H/^15^N-labeled ngMinE at 700 MHz and 35 °C. The *q* = 0.5 DMPC:DHPC bicelle concentration was 100 mM. DHPC, 6:0 1,2-dihexanoyl-sn-glycero-3-phosphocholine; DMPC, 14:0 1,2-dimyristoyl-sn-glycero-3-phosphocholine; ngMinE, *Neisseria gonorrhoeae* MinE.

To further probe the dynamics of bicelle-bound ngMinE and to ascertain the relative orientation of helix α1 to helix α2 on the surface of the membrane, we carried out ^15^N-relaxation measurements (^15^N-*R*_2_, ^15^N-*R*_1_ and ^15^N-{^1^H} NOE) on both free and bicelle-bound ngMinE (Figs. 3, S5 and S6; Tables S4 and S5). The ^15^N-*R*_2_ values were derived from ^15^N-*R*_1ρ_ measurements using a spin lock with a 2 kHz radiofrequency field which is sufficient, in the case of ngMinE, to suppress exchange line-broadening on the sub-millisecond to millisecond time scale.

Analysis of the ^15^N-relaxation data obtained for free full-length ngMinE, using standard procedures (27) based on the NMR coordinates of free ngMinE (PDB 6U6P) (14), yields values of 11.7 ± 0.1 ns and 1.8 ± 0.1 for the effective rotational correlation time, *τ*_*c*,*eff*_ = (2*D*_||_ + 4*D*_⊥_)^−1^, and diffusion anisotropy, *D*_||_/*D*_⊥_, respectively (Table 1), where *D*_||_ and *D*_⊥_ are the diffusion tensor constants parallel and perpendicular to the unique axis of the diffusion tensor. The polar and azimuthal angles, *θ* and *φ*, respectively, that define the orientation of the principal axis of diffusion (*D*_||_) in the molecular frame, have values of 90 ± 3° and −7 ± 2°, respectively, where the *C*_2_ symmetry axis of the ngMinE dimer lies along the *z*-axis of the coordinate system (14). Note that the principal axis of the diffusion tensor is orthogonal to the two-fold symmetry axis of the ngMinE dimer (*θ* = 90°). The agreement between observed and calculated relaxation parameters is excellent as shown by a comparison of the experimental points and calculated curves for ρ = *R*_1_/(2*R*_2_ − *R*_1_) (28) *versus* the angle α that the N-H bond vectors subtends to the unique axis of the diffusion tensor (Fig. 3*A*, top panel), as well as the correlation plot between experimental and calculated values of ρ (Fig. 3*B*, top panel). Moreover, the data for both the protein core and α1 helix for free ngMinE are fit using a single set of parameters indicating that helix α1 and the protein core tumble as a single unit.

**Table 1:** Parameters of the axially symmetric global rotational diffusion tensor for free and bicelle-bound ngMinE^a^

Diffusion parameters	Free ngMinE^b^	Bicelle-bound ngMinE
*τ*_*c*,*eff*_ (ns)	11.7 ± 0.05	27.5 ± 0.5
*D*_||_/*D*_⊥_	1.8 ± 0.05	2.2 ± 0.2
*θ*; *φ* (º)^c^	90 ± 3; −7 ± 2	90 ± 1; −18 ± 4
*θ′*; *φ′* (º)^c^	–	90 ± 9; −6 ± 6
τ_slow_ (ns)	–	1.5 ± 0.2
$S_{slow}^{2}$ (α1)^d^	_	0.7 ± 0.3
$S_{slow}^{2}$ (core)^d^	_	0.9 ± 0.1

a The uncertainties in the fitted parameters were determined from 200 Monte-Carlo simulations of the fits.

b Only amide sites belonging to secondary structure elements of the protein and having ^1^H-^15^N NOE > 0.4 were included in analyses of free and bicelle-bound ngMinE. The values of *τ*_*c*,*eff*_ and anisotropy *D*_||_/*D*_⊥_ for free MinE are somewhat higher than expected for a ∼19 kDa protein dimer with ratio of dimensions of ∼1.5 at 35 ºC and are likely the consequence of the presence of two unstructured (dangling) 12-residue C-terminal ends (residues 84–89 of ngMinE plus a His6 tag that was not deleted) located on opposite faces of the dimer. A similar phenomenon has been observed for various proteins bearing intrinsically disordered segments and tails (49, 50).

c The polar/azimuthal angle pairs (*θ′*; *φ′*) and (*θ*; *φ*) define the orientations of the unique axis of the rotational diffusion for the α1 helices in bicelle-bound ngMinE and the core of the ngMinE structure, respectively, in the molecular frame, where the *C*_2_ symmetry axis of the ngMinE dimer lies along the *z*-axis of the coordinate system (PDB 6U6P; (14)). The principal axis of the diffusion tensor is orthogonal to the *C*_2_ symmetry axis.

d The order parameter squared for fast N–H bond librations, $S_{fast}^{2}$, is fixed at 1. The protein core is defined as the six-stranded β-sheet and the α2 helices.

The analysis of the ^15^N-relaxation data for bicelle-bound ngMinE requires a more complex model that makes use of the extended Lipari-Szabo formalism (29), adapted to anisotropic global molecular reorientation (30). This is because the data for residues within the α1 helix and protein core cannot be accounted for simultaneously using the simple formalism used for free ngMinE. In the case of bicelle-bound ngMinE, the spectral density, *J*(*ω*) is given by:

(1) $$J\left(\omega \right)=\sum_{n=1}^{3}A_{n}\left\{S_{fast}^{2}S_{slow}^{2}\frac{\tau_{n}}{1+{\left(\omega \tau_{n}\right)}^{2}}+S_{fast}^{2}\left(1-S_{slow}^{2}\right)\frac{\tau_{slow,n}^{\prime }}{1+{\left(\omega \tau_{slow,n}^{\prime }\right)}^{2}}+\left(1-S_{fast}^{2}\right)\frac{\tau_{fast,n}^{\prime }}{1+{\left(\omega \tau_{fast,n}^{\prime }\right)}^{2}}\right\}$$

where

$$A_{1}=\left(3/4\right)\sin^{4}\;\alpha ;A_{2}=3\;\sin^{2}\;\alpha \cos^{2}\;\alpha ;A_{3}=\left(1/4\right){\left(3\;\cos^{2}\;\alpha -1\right)}^{2}$$

(2) $$\tau_{1}={\left(4D_{∥}+2D_{⊥}\right)}^{-1};\tau_{2}={\left(D_{∥}+5D_{⊥}\right)}^{-1};\tau_{3}={\left(6D_{⊥}\right)}^{-1}$$

$${\left(\tau_{slow,n}^{\prime }\right)}^{-1}={\left(\tau_{slow}\right)}^{-1}+{\left(\tau_{n}\right)}^{-1};{\left(\tau_{fast,n}^{\prime }\right)}^{-1}={\left(\tau_{fast}\right)}^{-1}+{\left(\tau_{n}\right)}^{-1}$$

α is the angle between the N-H bond vector and the unique axis of the rotational diffusion tensor; *τ*_slow_ and *τ*_fast_ are the timescales (correlation times) of slow and fast N-H bond vector motions, respectively; and *S*_slow_ and *S*_fast_ are the order parameters of the slow and fast motions, respectively. Note that no local (residue-specific) motional parameters are used in this model of dynamics.

During the initial stages of analysis of the bicelle-bound ngMinE ^15^N relaxation data, it became apparent that the dynamics parameters describing fast N-H bond vector librations ($S_{fast}^{2}$ and *τ*_fast_) cannot be determined with certainty; subsequent calculations were therefore performed with $S_{fast}^{2}$ in Equations 1 and 2 set to 1. Equations 1 and 2 can be re-cast in terms of the effective correlation time, *τ*_*c*,*eff*_ = (2*D*_||_ + 4*D*_⊥_)^−1^, and anisotropy of global rotational diffusion, *D*_||_/*D*_⊥_, which are used as variable parameters in the fit, together with (a) the time-scale of slow motions *τ*_slow_ (assumed to be the same for helix α1 and the protein core); (b) two separate values of $S_{slow}^{2}$, one for helix α1 and the other for the protein core (the relaxation data for helix α1 cannot be fit when a single value of $S_{slow}^{2}$ is used); and (c) two separate pairs of polar/azimuthal angles, (*θ′*, *φ′*) and (*θ*, *φ*), defining the orientations of the unique axes of the global rotational diffusion of the α1 helices and protein core, respectively, in the molecular frame where the *C*_2_ symmetry axis of the ngMinE dimer lies along the *z*-axis of the coordinate system (14). The rationale behind using two separate sets of polar/azimuthal angles for the α1 helices and protein core is based on the fact that the short α1 helices are connected to the rest of the ngMinE structure by long flexible linkers (Fig. 1*A*, left panel) and therefore could readily change orientation with respect to the protein core upon ngMinE binding to the bicelle surface.

The resulting global fits to the ^15^N-relaxation data (^15^N-*R*_2_, ^15^N-*R*_1_ and ^15^N-{^1^H} NOE) for bicelle-bound ngMinE are shown in Figure 3, *A* and *B* (bottom panels) and Fig. S6, and the values of the optimized parameters are summarized in Table 1. The reduced χ^2^ is 1.6. The values of the overall effective correlation time (*τ*_*c*,*eff*_) and diffusion anisotropy (*D*_||_/*D*_⊥_) are 27.5 ± 0.5 ns and 2.2 ± 0.2, respectively. The correlation time (*τ*_slow_) for slow rigid body motion on the surface of the bicelle is 1.5 ± 0.2 ns. The order parameter squared, $S_{slow}^{2}$, for slow (nanosecond time scale) rigid body motions of helix α1 is 0.7 ± 0.3 compared to 0.9 ± 0.1 for the protein core; in the context of a restricted diffusion model on the surface of the bicelles about an axis perpendicular to the membrane with a semi-angle *θ*, these $S_{slow}^{2}$ values would correspond to *θ* values of 34° and 18°, respectively (where $S_{slow}^{2}=1/4+\left(3/16\right){\left[\sin\left(2\theta \right)/\theta \right]}^{2}$ (31)).

**Figure 4:**
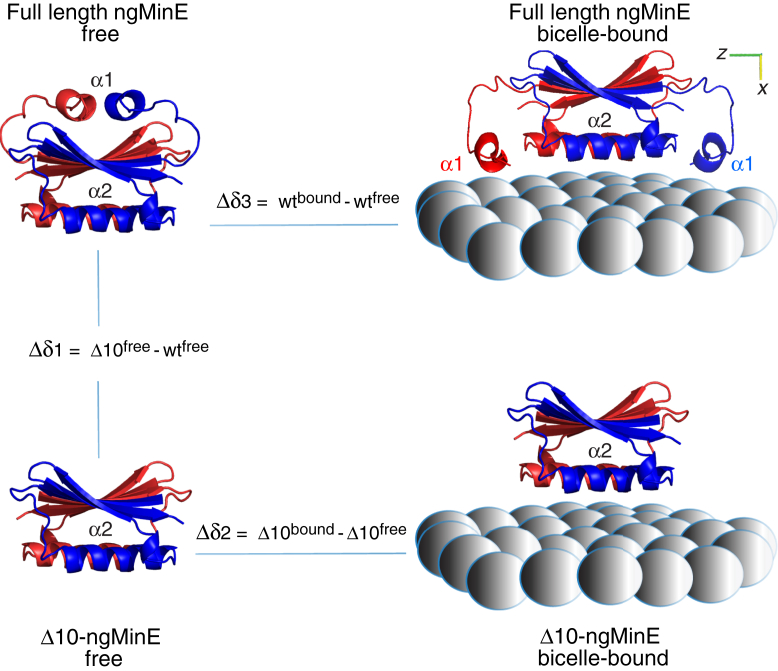
**Model of how full-length and Δ10 ngMinE are oriented on the surface of *q* = 0.5 DMPC:DHPC bicelles derived from analysis of PRE and**^**15**^**N relaxation data.** Differences in chemical shifts (Δδ1, Δδ2 and Δδ3) are defined in the figure. The surface of the bicelle is represented by the *gray spheres*. DHPC, 6:0 1,2-dihexanoyl-sn-glycero-3-phosphocholine; DMPC, 14:0 1,2-dimyristoyl-sn-glycero-3-phosphocholine; ngMinE, *Neisseria gonorrhoeae* MinE; PRE, paramagnetic relaxation enhancement.

Finally, the pair of polar/azimuthal angles are (90 ± 9°, −6±6°) for helix α1 compared to (90 ± 1° and −18± 4°) for the protein core, indicating that the orientation between the long axes of helices α1 and α2 while bound to the surface of the bicelle are only minimally perturbed relative to that seen in the structure of free ngMinE. This enables one to generate an approximate model of bicelle-bound ngMinE, depicted in Figure 4 (top), in which both helices α1 and α2 lie on the surface of the bicelle. The long axes of the α2 and α1 helices are approximately colinear and orthogonal, respectively, with the principal axis of rotational diffusion. Thus, while initial targeting of the membrane may occur *via* a previously identified excited state in which helix α1 is detached from the protein core and becomes disordered (14), thereby permitting the N-terminal membrane targeting region to potentially act a fly-cast, tight binding to the surface of the bicelle is obtained by the extensive interactions with the bicelle surface formed by both helices α1 and α2.

The value of the effective overall correlation time, *τ*_*c*,*eff*_, for bicelle-bound ngMinE is consistent with the molecular weight (∼70 kDa) of the complex at 35 °C. The anisotropy of rotational diffusion (*D*_||_/*D*_⊥_) as well as the orientation of the principal axis are very similar for free and bicelle-bound ngMinE (Table 1). Since *q* = 0.5 DMPC:DHPC bicelles are disc-like in shape (*i.e.*, oblate ellipsoids) (19) and therefore expected to tumble almost isotropically in solution (32), one can conclude that the values of *D*_||_/*D*_⊥_ and (*θ*, *φ*) are largely determined by ngMinE with the small differences between free and bicelle-bound ngMinE arising from binding to the much larger bicelle particle.

### ^1^H_N_/^15^N chemical shift changes within the protein core arising from helix α1 detachment and bicelle binding are additive

**Figure 5:**
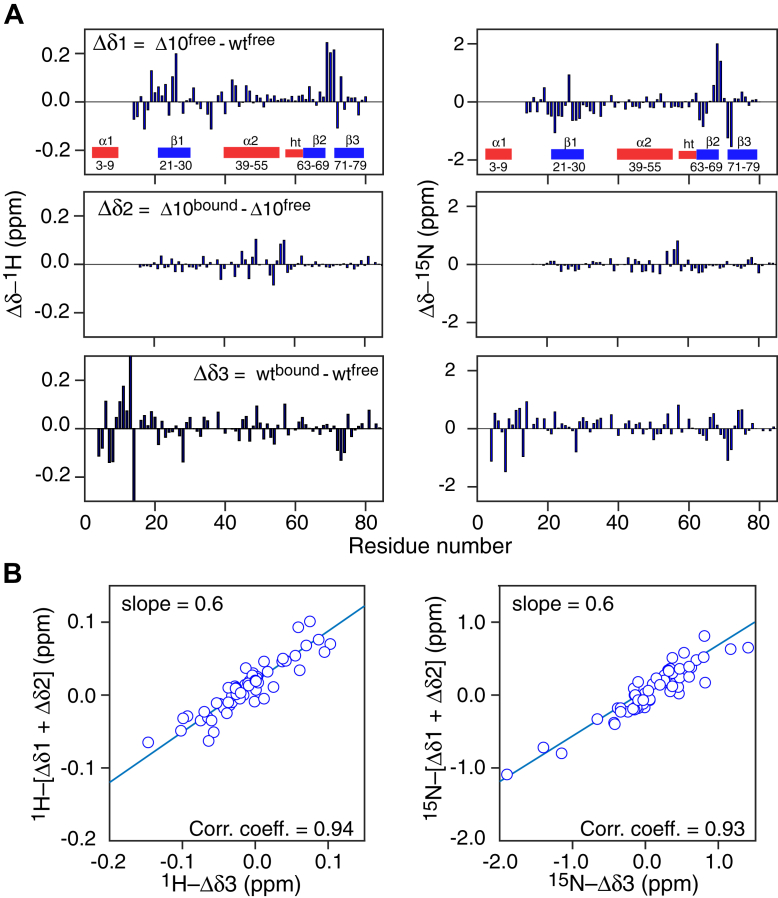
**Chemical shift perturbation data indicate that the core of full-length and Δ10 ngMinE interact in the same manner with *q* = 0.5 DMPC:DHPC bicelles.** The core of both proteins comprises the three β-strands and the α2 helix. *A*, ^1^H_N_ (*left*) and ^15^N (*right*) chemical shift differences between free full-length and Δ10 ngMinE (Δδ1, *top panel*), bicelle-bound and free Δ10 ngMinE (Δδ2, *middle panel*), and bicelle-bound and free full-length ngMinE (Δδ3, *bottom panel*). *B*, correlation of (Δδ1 + Δδ2) *versus* Δδ3 for ^1^H_N_ (*left*) and ^15^N (*right*) backbone atoms. Note that (Δδ1 + Δδ2) is equivalent to the chemical shift difference between Δ10 bound and full-length free. DHPC, 6:0 1,2-dihexanoyl-sn-glycero-3-phosphocholine; DMPC, 14:0 1,2-dimyristoyl-sn-glycero-3-phosphocholine; ngMinE, *Neisseria gonorrhoeae* MinE.

Although most marked for residues in helix α1, differences in ^1^H_N_ and ^15^N chemical shift differences between free and bicelle-bound ngMinE are spread throughout the protein sequence (Fig. 1*B*). To dissect these chemical shift changes upon bicelle binding, we compared the ^1^H_N_ and ^15^N chemical shift changes within the protein core between bicelle-bound and free full-length ngMinE (Δδ3) with those between the free Δ10 and full-length ngMinE constructs (Δδ1) and those between bicelle-bound and free Δ10-ngMinE (Δδ2) (Figs. 4 and 5*A*; Tables S1, S2, S6, and S7). There is a very strong correlation (correlation coefficient > 0.9) between Δδ1 + Δδ2 (which is equivalent to Δ10^bound^ – wt^free^) *versus* Δδ3 (wt^bound^ – wt^free^) for both ^1^H_N_ and ^15^N chemical shifts (Fig. 5*B*). One can therefore conclude that the changes in ^1^H_N_/^15^N chemical shifts within the protein core arising from helix α1 detachment and binding of helix α2 to the membrane are independent of one another and approximately additive.

### Relaxation dispersion and submillisecond global dynamics within bicelle-bound ngMinE

**Figure 6:**
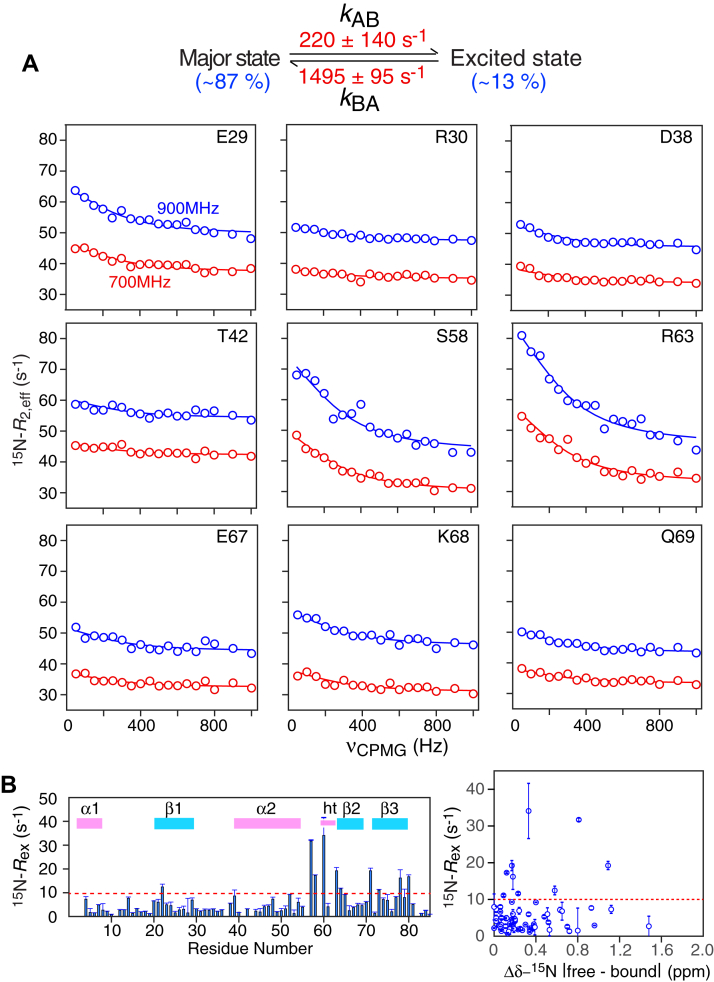
^**15**^**N CMPG relaxation dispersion demonstrates that bicelle-bound ngMinE undergoes global exchange dynamics.***A*, examples of ^15^N CPMG relaxation dispersion profiles recorded at 700 (*red*) and 900 (*blue*) MHz on 0.5 mM ^15^N/^2^H-labeled ngMinE in the presence of 100 mM *q* = 0.5 DMPC:DHPC bicelles (33 mM DMPC, 67 mM DHPC) at 35 °C. The experimental data are shown as *circles* (and the error are smaller than the diameter of the circles), and the best-fit curve to a two-state exchange model (with the rate constants listed at the *top* of the figure) are displayed as continuous lines. The values for the differences in ^15^N chemical shifts between major and minor species are provided in Table S8. *B*, plot of ^15^N-*R*_ex_ as a function of residue (*left panel*) and correlation between ^15^N-*R*_ex_ and the difference in ^15^N chemical shift between free and bicelle-bound ngMinE (*right panel*). The absence of any correlation indicates that the dynamics observed by CPMG relaxation dispersion on bicelle-bound ngMinE do not arise from exchange between the free and bound states. ^15^N-*R*_ex_ is the difference in effective ^15^N-*R*_2_ obtained at CPMG fields (v_CPMG_) of 0 and 1000 Hz. Error bars: 1 S.D.; ht, helical turn. CPMG, ^15^N Carr-Purcell-Meiboom-Gill; DHPC, 6:0 1,2-dihexanoyl-sn-glycero-3-phosphocholine; DMPC, 14:0 1,2-dimyristoyl-sn-glycero-3-phosphocholine; ngMinE, *Neisseria gonorrhoeae* MinE.

The backbone chemical shift, backbone amide-amide NOE, and backbone amide RDC data clearly indicate that the six-stranded β-sheet structure of the ngMinE core remains intact upon binding to the bicelle surface (Figs. 1*C*, S3 and S4). Free ngMinE, however, experiences submillisecond to millisecond global dynamics resulting in the population of sparsely populated excited states that may represent intermediates on the transition path between the resting six-stranded and active four-stranded states (14). ^15^N Carr-Purcell-Meiboom-Gill (CPMG) relaxation dispersion measurements on bicelle-bound ngMinE also reveal evidence of exchange (Fig. 6). The ^15^N relaxation dispersion data were globally fit to a simple two-state exchange model between a major ground-state species populated at ∼87% and an excited state populated at ∼13% with an exchange lifetime of ∼600 μs, which lies on the fast side of intermediate exchange on the chemical shift time scale. (Note that exchange line broadening is largely suppressed at a 1 kHz CPMG field, and hence the ^15^N-*R*_2_ relaxation data presented in Figure 3 and Fig. S6 are not ‘contaminated’ by exchange line-broadening, *R*_ex_, as these data were derived from ^15^N-*R*_1ρ_ measurements acquired with a 2 kHz radiofrequency field). It is also worth noting that the analysis of the rotational diffusion tensor of bicelle-bound ngMinE, presented above, implicitly assumes that the diffusion properties of the minor species are very similar to those of the major state. Indeed, substantial changes in the rotational diffusion tensor are unlikely to occur as both species are bound to the surface of bicelles. Hence, similar intrinsic (exchange-free) ^15^N-*R*_2_ rates are expected for the interconverting major and minor states, with analysis of bicelle-bound ngMinE diffusion not compromised appreciably by exchange.

While we could only quantitatively analyze the dispersion curves for the 22 residues listed in Table S8 (sufficient to derive exchange parameters), a plot of the ^15^N-*R*_ex_ (difference in effective ^15^N-*R*_2_ between 0 and 1 kHz CPMG field) as a function of residue provides a measure of the differences in ^15^N chemical shift between the ground and excited states. The largest (>10 s^−1^) ^15^N-*R*_ex_ values are observed in the loop connecting helix α2 and the helical turn, as well as for a few residues within the β2 and β3 strands and one in strand β_1_ (Fig. 5*B*, left panel). Given that the ^15^N-*R*_ex_ values are uncorrelated with the absolute differences in ^15^N chemical shifts between free and bicelle-bound ngMinE, one can conclude that the exchange phenomenon observed by CPMG relaxation dispersion relates entirely to the interconversion between bicelle-bound ground and excited states. Given the location of the largest ^15^N-*R*_ex_ values noted above, it is tempting to speculate that the excited state may represent a very early intermediate along the pathway between six- and four-stranded states of ngMinE. The complete transition from six-to four-stranded forms involves a large structural rearrangement entailing extrusion of the β_1_ strands from the dimer interface and their replacement by the β_2_ strands (see Fig. 1*A*). It is also worth noting that no significant *R*_ex_ values are observed for residues within helix α1 indicating that exchange on the submillisecond time scale between ground and excited states reported by relaxation dispersion is independent of the nanosecond rigid body motions on the surface of the membrane reported on by the ^15^N-*R*_1_ and *R*_2_ relaxation data (Table 1, Figs. 3 and S6).

### Concluding remarks

Here we have investigated the structure and dynamics of ngMinE bound to *q* = 0.5 DMPC:DHPC bicelles using a variety of NMR methods. The interaction of the ngMinE dimer with the bicelle surface involves two distinct points of contact involving the α1 and α2 helices, oriented approximately orthogonal to one another (Fig. 4). Helix α1, which is connected to the protein core by a long linker, experiences approximately 2-fold larger amplitude restricted diffusion on the surface of the bicelles about an angle perpendicular to the membrane surface relative to the protein core.

Taken together with previous ^15^N CPMG relaxation measurements on free ngMinE (14), one can conclude that ngMinE binding to the bicelle surface may occur *via* the previously identified excited state in which helix α1 is disordered and no longer attached to the underlying six-stranded β-sheet (14), such that the N-terminal region may potentially act as a fly-cast to provide the initial point of attachment to the bicelle surface, followed by binding of the protein core *via* the two α2 helices. The bicelle-bound state of ngMinE, however, does experience exchange between the major ground state and a minor excited state on a time scale of ∼600 μs, and it is tempting to speculate that this exchange phenomenon may entail a very early intermediate along the transition pathway between the resting six-stranded and active four-stranded conformations of membrane-bound ngMinE.

## Experimental procedures

### Protein expression and purification

Full-length *N*. *gonorrhoeae* ngMinE bearing the double mutation E46A/R10Q, designed to improve solubility and resistance to proteolytic cleavage, respectively, as well as the 10-residue N-terminal deletion Δ10-ngMinE, was purified, expressed, and isotopically labeled as described previously (14, 33). Two isotope labeling schemes were used: uniform ^2^H/^15^N and ^2^H/^15^N/^13^C labeling. The full-length and Δ10 ngMinE constructs comprise residues 1 to 89 and 11 to 89, respectively, of ngMinE followed by a (His)_6_ tag that was not deleted.

### *q* = 0.5 DMPC:DHPC bicelle preparation

DMPC and DHPC were purchased as powders from Avanti Polar lipids. The 300 mM total lipid concentration stock solution of *q* = 0.5 DMPC:DHPC bicelles was prepared as follows. 100 mg DHPC was dissolved in 1.1 ml buffer (25 mM potassium phosphate, pH 6.5, 1 mM ethylenediaminetetraacetic acid, 1 mM benzamidine hydrochloride, 95% v/v H_2_O/5% v/v D_2_O) to yield a 200 mM DHPC solution. 75 mg DMPC was then added, corresponding to 100 mM DMPC. The *q* = 0.5 DMPC:DHPC mixture was left at room temperature for a few hours with occasional gentle vortexing and then placed in a 4 °C refrigerator overnight. If the bicelle stock was not homogeneous after overnight refrigeration, it was subject to several freeze/thaw cycles before storage at −80 °C.

### NMR sample preparation

All NMR samples and bicelle stock solution were prepared in 25 mM potassium phosphate pH 6.5, 1 mM EDTA, 1 mM benzamidine hydrochloride, and 95% (v/v) H_2_O/5% (v/v) D_2_O.

### NMR experiments

All NMR experiments were recorded at 35 °C on Bruker 500, 600, 700, 800, or 900 MHz spectrometer equipped with *z*- or *x*,*y*,*z*-gradient triple resonance cryoprobes. Backbone resonance assignments were obtained from 3D scalar through-bond correlation experiments (HNCA, HNCN, HNCOCA, HNCOCB, and HNCO) (34). Spectra were processed using the software package NMRPipe (35) and analyzed using the program XIPP (36). A 150 ms mixing time TROSY-based 3D ^15^N-separated NOE experiment (NOE-TROSY) was acquired on a ^2^H/^15^N-labeled sample with nonuniform sampling, comprising 36.1% of the full data matrix of 95∗ (^1^H *t*_1_) × 100∗ (^15^N *t*_2_) × 1536∗ (^1^H *t*_3_) complex points with acquisition times of 10.3, 45.2, and 104.4 ms, respectively, and reconstructed using SMILE (37).

### Backbone ^15^N relaxation experiments

^15^N-*R*_1_, *R*_1ρ_ and ^15^N-{^1^H} NOE data were collected at 700 MHz using TROSY-based ^1^H-^15^N correlation experiments (38). The radiofrequency field strength used for the *R*_1ρ_ measurements was 2 kHz which is sufficient to suppress line broadening arising from chemical exchange, for both free (14) and bicelle-bound (see CPMG dispersion profiles in Fig. 6) ngMinE. The *R*_1_ and *R*_1ρ_ data were acquired with 80∗ × 1200∗ complex points in the ^15^N (t_1_) and ^1^H (t_2_) dimensions (24 scans per increment), respectively, with corresponding acquisition times of 37.5 and 102 ms; the data for the different spin-lock or relaxation delays (see below) were acquired in an interleaved manner. ^15^N-*R*_2_ values were obtained from the *R*_1ρ_ and *R*_1_ values using the equation (*R*_1ρ_ − *R*_1_cos^2^*θ*)/sin^2^*θ*, where *θ* is the angle between the effective spin-lock field and the external magnetic (*B*_o_) field. The *R*_1ρ_ values were obtained by fitting a single exponential to 7 (1, 5, 10, 20, 40, 60, and 80 ms) and 8 (1, 2.5, 5, 10, 15, 20, 30, and 40 ms) spin-lock times for free and bicelle-bound ngMinE. Likewise, *R*_1_ values were obtained by fitting a single exponential to 6 (40, 400, 800, 1200, 1600, and 2000 ms) and 5 (40, 400, 800, 1200, and 1600 ms) relaxation delays for free and bicelle-bound ngMinE. For the ^15^N-{^1^H} NOE measurements (39), two datasets were recorded in an interleaved manner with and without 8 s ^1^H saturation (with a hard 180° pulse train applied at intervals of 22 ms), as described in (25, 38) (in the absence of ^1^H saturation the interscan delay is set equal to the ^1^H saturation time); 64 scans were recorded per increment with 80∗ and 1200∗ complex points in the ^15^N (t_1_) and ^1^H (t_2_) dimensions, respectively, and corresponding acquisition times of 37.5 and 102 ms.

### PRE measurements

Backbone amide proton transverse PRE rates (^1^H_N_-Γ_2_) on bicelle-bound full-length and Δ10 ngMinE were recorded by taking the difference in ^1^H-*R*_2_ rates measured in the presence and absence of one or 2 mM nitroxide (16-DSA) incorporated into the bicelles (24, 25). ^1^H-*R*_2_ measurements were carried out at 800 MHz as described previously (40, 41).

### Measurement of backbone amide RDCs in stretched gels

Backbone amide ^1^*D*_NH_ RDCs were measured at 700 MHz as the difference in one-bond N-H couplings in aligned (stretched gel) and isotopic (water) media using the TROSY-based ARTSY method (42). Alignment was obtained using a 4.5% positively charged stretched gel comprising acrylamide (4.16% w/v), bisacrylamide (0.11% w/v), and 3-acrylamidopropyl-trimethylammonium chloride (0.23% w/v) (43). The cast gel was first soaked in 50 ml buffer comprising 100 mM sodium phosphate, pH 7.4 overnight, and then soaked in milliQ-purified H_2_O twice (overnight or for a minimum of 4 h) before drying. To prepare the sample of bicelle-bound ngMinE in the gel, the dried gel was soaked in a 320 μl sample of 0.5 mM (in subunits) ngMinE, 100 mM *q* = 0.5 DMPC:DHPC, 25 mM potassium phosphate pH 6.5, 1 mM EDTA, and 1 mM benzamidine hydrochloride. The gel was then radially compressed from 4.9 to 4.2 mm in diameter using a funnel for insertion of the gel into the NMR tube (44).

### ^15^N CPMG relaxation dispersion measurements

^15^N-CPMG experiments were recorded, as described previously for free ngMinE (14), at 700 and 900 MHz on bicelle-bound ngMinE using a pulse scheme that quantifies the relaxation rates of in-phase ^15^N coherences (45). The relaxation period was set to 20 ms, and the CPMG field strengths (ν = 1/2τ_CP_, where τ_CP_ is the time between two consecutive 180° pulses of the CPMG train) were 50, 100, 150, 200, 250, 300, 350,400, 500, 550, 600, 650, 700, 750, 800, 900, and 1000 Hz. Continuous wave decoupling of amide protons during the relaxation period was carried out using a nominal ^1^H radiofrequency field strength of 11 kHz, adjusted for each value of ν as described in ref. (45). Uncertainties in *R*_2,eff_ values were obtained from duplicate measurements at one ν_CPMG_ value (45).

The residues included for quantitative analysis of the relaxation dispersion curves were restricted to those with large *R*_ex_ value (see Fig. 6) and this was sufficient to extract exchange parameters. Residues not included in the fit consisted of cross-peaks that were either too weak (resulting in poor signal-to-noise), exhibited chemical shift overlap or only displayed very small dispersions. The ^15^N relaxation dispersion curves at 700 and 900 MHz for all the residues listed in Table S8 were fit simultaneously, using an in-house script written in Matlab, to a two-state exchange model by numerically solving the appropriate McConnell equations, optimizing the forward and backward rate constants as global parameters, and the transverse relaxation rates and differences in chemical shifts for each residue as described in (14). The standard deviation of the optimized parameters was obtained from the variance-covariance matrix.

### ^15^N relaxation data analysis

The analysis of the rotational diffusion tensor of free ngMinE from ^15^N amide relaxation rates was performed using in-house scripts written in Matlab and verified with the Modelfree (27) and ROTDIF (28) programs. All these methods provided essentially identical results (see Table 1). The analysis of bicelle-bound ngMinE was carried out with in-house scripts on ^15^N *R*_2_, *R*_1_ and ^1^H-^15^N NOE data collected at 700 MHz using the form of the spectral density function in Equations 1 and 2 (see text for details). Only the amide sites belonging to secondary structure elements of the protein and having ^1^H-^15^N NOE >0.4 were included in analyses of both forms of ngMinE. The uncertainties in the fitted parameters were determined from 200 Monte-Carlo simulations of the fits (46).

### Modeling of ngMinE on the bicelle surface

To calculate a model of membrane-bound ngMinE, Xplor-NIH (47) was used with input coordinates of free ngMinE (PDB ID 6U6P) where the *z* axis is the *C*_2_ symmetry axis. In this calculation, the protein core (residues 21–81) was fixed in space, the α1 helix (residues 1–10) coordinates moved as a rigid body, and the linker residues 11 to 22 were given torsion angle or Cartesian degrees of freedom. The linker residues were given random torsion angles at the beginning of each calculation consisting of molecular dynamics simulated annealing on an energy surface consisting of a sum of terms. A planarity energy term restrained the Cα atoms of the α1 helix within 4 Å of the average *z*-value of the Cα atoms of the α_2_ helices (residues 41–54). A Python-based energy term was used to restrain the angles between the long axes of the α_1_ and α_2_ helices to correspond to the 12° difference between the azimuthal angles *φ and φ′* of the diffusion tensor, determined from analysis of the ^15^N relaxation data (see Table 1). Symmetry restraints were used so that the resulting structures have *C*_2_ symmetry. The following additional energy terms were used in the calculation: HBPot, RepelPot, TorsionDBPot, bonds, bond angles, and improper dihedral angles (47).

## Data availability

All discussed experimental data are included in the article and/or Supporting information. Raw NMR data and pulse sequences are available upon request to the authors.

## Supporting information

This article contains supporting information (14, 23, 42, 47).

## Conflict of interest

The authors declare that they have no conflicts of interest with the contents of this article.
