# Supplementary material for: Interaction of the bacterial division regulator MinE with lipid bicelles studied by NMR spectroscopy
Source: J Biol Chem. 2023 Feb 17;299(4):103037. doi: 10.1016/j.jbc.2023.103037 (PMC10031476; doi:10.1016/j.jbc.2023.103037)
Supplement: Supporting Figures S1–S6 and Tables S1–S8 [file mmc1.pdf]

SUPPORTING INFORMATION

Interaction of the bacterial division regulator MinE with lipid bicelles studied by NMR spectroscopy

Mengli Cai,[a] Vitali Tugarinov,[a] Sai C. Chiliveri,[a] Ying Huang,[b] Charles D. Schwieters,[a,c] Kyoshi Mizuuchi[b] and G. Marius Clore[a,d]

[a]Laboratory of Chemical Physics, [b]Laboratory of Molecular Biology and [c]Computational Biomolecular Magnetic Resonance Core, National Institute of Diabetes and Digestive and Kidney Diseases, National Institutes of Health, Bethesda, MD 20892-0520, U.S.A.

[d]Author to whom correspondence should be addressed: G.M.C. mariusc@mail.nih.gov

Contents:

8 SI Tables
6 SI Figures

Table S1. Backbone chemical shift (ppm) assignments for free ngMinE at 35ºC

Residue	$^{1}H_{N}$	^{15}N	$^{13}C\alpha$	$^{13}C\beta$	$^{13}C'$
S2	8.20	121.49	57.93	64.06	-
L3	-	-	57.67	41.71	178.84
I4	8.40	119.08	65.43	37.05	177.37
E5	7.79	118.85	59.23	29.27	178.94
L6	7.54	119.17	57.45	41.92	178.56
L7	7.99	116.78	56.74	41.62	178.74
F8	8.11	116.32	58.31	39.06	177.27
G9	7.91	108.10	46.32	-	174.70
Q10	8.03	118.71	56.31	28.75	176.56
M11	8.03	121.06	57.13	32.27	176.90
Q12	8.26	119.89	56.30	29.12	176.39
K13	8.37	122.80	-	32.69	176.83
T14	8.02	113.42	61.83	70.02	174.03
T16	-	-	-	69.99	174.30
V17	8.01	122.02	62.03	32.55	175.45
A18	8.37	128.41	52.65	19.24	176.90
R19	7.97	119.81	54.74	32.19	174.50
D20	8.26	121.07	54.59	41.64	175.78
R21	8.77	120.20	55.64	34.00	173.93
L22	8.77	120.43	53.79	46.62	174.93
Q23	9.39	125.43	54.79	32.52	173.87
I24	9.15	124.53	-	40.87	174.06
I25	9.56	127.61	59.69	41.04	174.61
I26	9.38	125.41	60.42	39.80	173.24
A27	9.47	128.70	50.11	22.21	175.30
Q28	9.33	123.56	54.71	33.50	173.60
E29	8.94	128.81	55.28	32.75	174.79
R30	8.37	123.46	54.38	33.10	175.73
A31	8.75	126.24	52.64	18.88	178.85
Q32	8.69	120.39	57.21	29.18	176.58
E33	8.68	119.07	57.30	29.05	176.88
G34	7.96	107.77	45.59	-	174.35
Q35	8.27	116.99	55.87	29.31	175.90
T36	7.99	119.23	60.99	69.71	-
P37	-	-	62.86	31.75	173.14
D38	7.82	116.34	53.09	40.61	177.63
Y39	9.58	125.86	59.98	39.01	176.69
L40	8.57	122.04	61.17	38.03	-
P41	-	-	-	30.73	179.93
T42	7.33	113.86	66.20	68.78	176.43
L43	8.34	121.29	57.92	41.01	177.88
R44	8.97	119.64	60.48	29.74	177.07
K45	7.31	117.16	59.71	32.03	179.15
A46	7.89	120.14	54.70	18.66	180.71
L47	8.70	117.64	57.21	40.59	178.72
M48	8.06	117.47	58.31	30.47	179.20
E49	7.58	119.41	59.64	29.08	179.08
V50	7.92	118.89	66.13	31.63	178.13
L51	8.64	119.40	58.17	40.89	178.84

Table S1 (cont.)

Residue	$^{1}H_{N}$	^{15}N	$^{13}C\alpha$	$^{13}C\beta$	$^{13}C'$
S52	7.90	112.87	61.39	63.12	176.03
K53	7.68	118.51	58.62	32.75	178.03
Y54	8.03	113.21	59.82	39.47	175.91
V55	7.83	117.45	61.64	31.82	174.72
N56	7.74	117.47	54.17	37.18	174.24
V57	7.34	114.76	60.12	33.65	174.75
S58	8.46	118.73	57.58	64.25	175.98
L59	8.55	123.10	57.87	40.96	178.49
D60	8.24	115.44	55.96	40.06	176.64
N61	7.94	116.34	53.71	39.80	173.60
I62	7.45	119.39	61.56	39.30	174.39
R63	8.64	127.46	55.34	31.45	175.74
I64	8.76	126.44	60.81	40.19	175.70
S65	9.14	122.69	57.39	65.41	171.94
Q66	8.54	122.71	55.06	31.89	174.69
E67	8.93	122.86	55.30	34.54	174.50
K68	8.63	123.27	55.79	35.29	175.54
Q69	8.74	124.17	55.73	31.36	174.50
D70	9.06	125.01	56.58	39.98	175.86
G71	8.55	109.80	46.12	-	173.40
M72	8.21	121.00	54.79	35.20	173.59
D73	8.74	123.99	53.90	45.35	174.69
V74	8.85	120.34	61.08	35.18	173.60
L75	9.35	129.42	53.56	45.81	173.36
E76	9.29	126.26	54.57	32.85	174.49
L77	9.61	125.83	54.29	43.43	175.15
N78	9.06	121.92	53.50	41.94	174.28
I79	9.11	125.38	60.77	40.54	175.05
T80	8.62	124.39	62.77	69.29	173.72
L81	8.03	127.39	53.13	39.85	-
P82	-	-	63.14	31.94	176.55
E83	8.53	121.47	56.74	30.26	176.66
Q84	8.58	122.89	55.78	29.25	175.80

Table S2. Backbone chemical shift assignments (ppm) for bicelle-bound ngMinE at 35°C

Residue	$^{1}H_{N}$	^{15}N	$^{13}C\alpha$	$^{13}C\beta$	$^{13}C'$
S2	8.25	121.69	57.83	64.04	-
L3	-	-	57.81	41.53	178.51
I4	8.29	117.78	64.51	37.05	177.41
E5	7.71	119.25	59.07	29.29	177.62
L6	7.64	119.37	57.38	41.71	178.55
L7	7.85	116.51	56.76	41.72	178.11
F8	7.96	114.85	58.02	39.23	177.05
G9	7.95	108.38	46.05	-	174.44
Q10	8.13	118.78	56.11	29.01	176.39
M11	8.20	121.63	56.79	32.35	176.81
Q12	8.33	120.51	56.01	29.03	176.21
K13	8.29	121.91	56.70	32.51	176.79
T14	8.01	114.58	61.74	69.85	174.12
A15	8.26	125.90	52.63	18.95	177.63
T16	8.09	113.92	61.90	69.87	174.33
V17	8.05	122.22	61.98	32.56	175.43
A18	8.38	128.34	52.55	19.18	176.86
R19	8.03	119.96	54.77	32.06	174.62
D20	8.30	121.01	54.67	41.68	175.70
R21	8.69	120.03	55.47	34.02	174.07
L22	8.80	121.04	53.71	46.27	174.76
Q23	9.36	125.34	54.77	32.51	173.97
I24	9.12	124.52	59.99	40.77	174.19
I25	9.55	127.61	59.67	40.79	174.76
I26	9.37	125.47	60.38	39.79	173.40
A27	9.44	128.57	50.08	22.65	175.45
Q28	9.20	122.70	54.71	33.08	173.95
E29	8.96	128.85	55.47	32.21	174.92
R30	8.41	123.78	54.48	32.61	175.72
A31	8.74	126.39	52.64	18.90	178.71
Q32	8.67	120.20	57.03	29.08	176.49
E33	8.65	119.21	57.18	29.01	176.97
G34	8.01	108.00	45.63	-	174.32
Q35	8.29	117.26	55.78	29.29	175.88
T36	8.01	118.15	60.69	69.61	-
P37	-	-	62.74	31.73	173.64
D38	7.89	116.82	53.27	40.65	177.47
Y39	9.58	125.64	59.81	38.93	176.91
L40	8.55	121.80	60.93	37.92	-
P41	-	-	66.59	30.66	179.89
T42	7.33	113.90	66.19	68.62	176.37
L43	8.32	121.45	57.86	41.02	178.01
R44	8.92	119.42	60.49	29.69	177.22
K45	7.35	117.28	59.61	32.03	179.03
A46	7.94	120.26	54.79	18.50	180.37
L47	8.64	117.35	57.25	40.71	178.79
M48	8.06	117.28	58.48	30.83	178.90
E49	7.67	119.53	59.54	29.04	179.17
V50	7.94	118.40	66.06	31.56	178.23

Table S2 (cont.)

Residue	$^{1}H_{N}$	^{15}N	$^{13}C\alpha$	$^{13}C\beta$	$^{13}C'$
L51	8.60	119.06	58.17	40.91	178.78
S52	7.92	112.52	61.23	63.07	175.88
K53	7.64	118.54	58.45	32.61	177.80
Y54	7.96	113.71	59.69	39.54	175.89
V55	7.84	117.10	61.46	32.04	-
N56	-	-	53.90	37.31	174.41
V57	7.44	115.62	60.36	33.52	174.84
S58	8.42	118.47	57.48	64.12	-
L59	-	-	-	41.44	-
D60	8.23	115.65	55.81	40.13	176.59
N61	7.97	116.29	53.65	39.72	173.64
I62	7.50	119.30	61.20	39.50	174.42
R63	8.64	127.10	55.25	31.73	175.49
I64	8.76	126.07	60.58	40.32	175.49
S65	9.12	122.22	57.26	65.42	172.11
Q66	8.55	122.94	55.22	31.59	174.58
E67	8.90	123.26	55.15	33.82	174.55
K68	8.64	123.34	55.67	34.30	175.93
Q69	8.73	123.89	55.16	31.00	174.86
D70	9.05	124.58	56.36	39.91	175.94
G71	8.55	108.62	45.99	-	173.62
M72	8.10	120.23	54.90	35.25	174.05
D73	8.63	123.84	54.23	44.42	174.83
V74	8.75	120.92	61.27	34.94	173.91
L75	9.41	129.93	53.58	45.41	173.63
E76	9.24	125.91	54.65	32.51	174.50
L77	9.58	125.67	54.39	43.43	175.02
N78	9.06	122.08	53.39	41.96	174.31
I79	9.13	125.27	60.65	40.63	175.09
T80	8.61	124.02	62.55	69.31	173.77
L81	8.11	127.27	53.13	39.92	-
P82	-	-	62.99	31.87	176.60
E83	8.55	121.40	56.77	30.19	176.70
Q84	8.58	122.90	55.67	29.22	175.86

Table S3. Experimental backbone amide $^{1}D_{NH}$ RDC values obtained for bicelle-bound ngMinE

Residue	RDC (Hz).	Error (Hz)
5	1.25	0.05
6	1.72	0.02
7	4.07	1.05
8	3.51	1.03
9	-2.12	0.14
10	2.51	0.16
11	1.31	1.54
12	2.16	0.09
13	-0.22	0.10
14	4.11	1.91
16	2.52	0.18
17	4.95	0.15
18	3.65	0.02
19	-2.55	0.08
20	-1.14	0.99
24	9.22	0.35
25	15.04	0.10
26	9.85	0.04
28	7.63	1.34
29	9.55	0.22
30	0.49	0.09
32	2.76	0.20
33	2.94	0.06
35	1.72	0.05
38	8.10	0.35
40	-4.70	0.07
42	-10.11	0.75
43	-1.50	0.05
44	-2.12	0.19
45	-8.85	0.62
46	-4.57	1.88
47	-1.75	1.71
48	-3.87	1.00
50	-6.11	0.24
52	0.39	0.47
53	-9.98	0.16
55	1.91	0.50
58	-5.01	0.02
60	-2.60	0.48
61	-0.10	0.42
63	8.66	2.46
64	7.99	0.55
65	11.25	0.58
67	10.35	0.50
68	4.71	0.83
69	2.78	0.06
70	5.30	0.02
71	4.51	0.13
72	0.01	0.63
74	10.24	0.50
75	9.67	0.95
76	13.49	1.48
78	6.42	0.45
79	8.66	1.52
81	3.29	0.51
83	8.26	0.31

Table S4. ^{15}N-R_1 (s^{-1}), ^{15}N-R_2 (s^{-1}) and ^{15}N-{^{1}H} NOE relaxation data for free ngMinE recorded at 700 MHz and 35°C.

Residue	R1	R1 err	R2	R2 err	NOE	NOE err
4	1.01	0.09	16.57	0.73	0.60	0.03
5	1.01	0.03	19.01	1.09	0.67	0.03
6	1.02	0.04	16.77	0.40	0.70	0.03
7	1.05	0.09	18.03	1.78	0.65	0.03
8	1.01	0.05	17.13	0.84	0.67	0.03
9	1.14	0.10	12.78	0.65	0.55	0.03
11	1.25	0.11	9.78	1.37	0.44	0.02
13	1.31	0.12	5.48	0.15	0.23	0.01
17	1.24	0.13	6.02	0.20	0.22	0.01
18	1.24	0.12	7.89	0.26	0.33	0.02
19	1.11	0.08	11.20	0.32	0.47	0.02
21	1.00	0.04	16.18	0.45	0.75	0.04
22	0.96	0.04	16.69	0.60	0.80	0.04
24	0.91	0.04	16.50	1.01	0.85	0.04
25	0.98	0.04	15.43	0.74	0.89	0.04
27	0.97	0.05	15.09	0.42	0.89	0.04
28	0.91	0.05	17.14	0.39	0.83	0.04
29	1.00	0.05	15.82	0.33	0.82	0.04
30	0.94	0.03	16.05	0.56	0.76	0.04
32	1.03	0.10	14.96	0.68	0.60	0.03
33	1.05	0.09	13.75	0.54	0.50	0.02
34	1.05	0.11	10.47	0.54	0.46	0.02
35	1.09	0.10	10.50	0.40	0.48	0.02
38	0.90	0.05	15.62	0.46	0.75	0.04
40	0.83	0.04	21.01	0.55	0.79	0.04
42	0.78	0.05	19.18	0.85	0.79	0.04
43	0.87	0.04	19.28	0.92	0.84	0.04
44	0.78	0.04	22.06	0.97	0.83	0.04
45	0.75	0.03	22.35	1.04	0.82	0.04
46	0.85	0.05	21.89	1.30	0.86	0.04
47	0.82	0.04	20.56	0.53	0.83	0.04
48	0.80	0.03	19.87	1.38	0.80	0.04
49	0.79	0.02	22.06	0.90	0.83	0.04
50	0.83	0.04	19.43	0.71	0.83	0.04
51	0.83	0.03	21.83	0.83	0.81	0.04
52	0.77	0.03	20.68	0.53	0.78	0.04
53	0.79	0.03	21.23	0.88	0.77	0.04
54	0.85	0.05	16.81	0.69	0.73	0.04
55	0.82	0.02	18.45	0.87	0.75	0.04
57	0.89	0.04	16.24	0.60	0.58	0.03
58	0.95	0.09	15.09	0.51	0.47	0.02
60	1.03	0.11	16.10	0.45	0.64	0.03
61	1.12	0.11	14.01	0.44	0.67	0.03
62	1.04	0.04	14.24	0.34	0.66	0.03
63	0.98	0.04	14.12	0.35	0.76	0.04
64	0.93	0.08	15.70	0.37	0.77	0.04
65	0.96	0.04	15.69	0.81	0.75	0.04
66	0.92	0.10	15.98	0.48	0.77	0.04
68	0.94	0.07	15.10	0.42	0.62	0.03
70	1.08	0.11	13.63	1.09	0.66	0.03
71	1.13	0.11	13.67	0.76	0.66	0.03
74	0.92	0.05	16.20	0.83	0.80	0.04
75	0.96	0.04	15.88	0.44	0.88	0.04

Table S4 (cont.)

Residue	R1	R1 err	R2	R2 err	NOE	NOE err
76	0.97	0.04	16.07	0.55	0.84	0.04
78	0.90	0.02	16.02	0.50	0.80	0.04
79	0.93	0.04	15.61	0.64	0.82	0.04
80	0.96	0.05	15.87	0.33	0.75	0.04
81	1.00	0.04	14.06	0.21	0.72	0.04
83	1.05	0.10	12.40	0.30	0.46	0.02
84	1.16	0.12	9.38	0.23	0.35	0.02

Table S5. ^{15}N-R_1 (s^{-1}), ^{15}N-R_2 (s^{-1}) and ^{15}N-{^{1}H} NOE relaxation data for bicelle-bound ngMinE recorded at 700 MHz and 35°C

Residue	R1	R1 err	R2	R2 err	NOE	NOE err
4	1.08	0.12	13.18	3.55	0.39	0.02
5	1.06	0.06	33.12	0.52	0.60	0.03
6	1.00	0.06	28.80	1.28	0.65	0.03
7	1.03	0.12	30.19	4.98	0.63	0.03
8	1.01	0.11	28.14	7.06	0.49	0.02
9	1.15	0.08	15.92	0.32	0.48	0.02
10	1.20	0.16	13.61	0.39	0.41	0.02
12	1.26	0.14	13.50	0.36	0.27	0.01
14	1.14	0.05	16.06	4.82	0.26	0.01
16	1.11	0.14	8.79	0.27	0.08	0.00
17	1.18	0.15	11.12	0.25	0.13	0.01
18	1.17	0.15	13.38	0.28	0.25	0.01
19	1.01	0.09	23.22	0.41	0.42	0.02
22	0.69	0.05	44.73	2.43	0.75	0.04
24	0.71	0.06	40.77	2.28	0.74	0.04
25	0.71	0.06	37.23	3.05	0.77	0.04
27	0.74	0.04	34.85	1.07	0.76	0.04
28	0.67	0.06	42.14	6.21	0.73	0.04
30	0.76	0.03	38.90	1.19	0.66	0.03
31	0.86	0.06	30.33	0.32	0.60	0.03
33	0.91	0.11	26.90	0.52	0.40	0.02
34	0.94	0.12	17.10	0.72	0.36	0.02
35	0.96	0.11	21.15	1.24	0.41	0.02
36	0.85	0.08	29.28	0.68	0.42	0.02
38	0.74	0.06	36.70	0.63	0.64	0.03
40	0.62	0.07	34.40	5.94	0.74	0.04
42	0.59	0.04	46.32	3.11	0.72	0.04
44	0.60	0.03	59.80	12.11	0.80	0.04
45	0.55	0.06	57.62	3.71	0.81	0.04
46	0.60	0.08	43.93	5.79	0.73	0.04
47	0.63	0.06	43.71	4.45	0.77	0.04
50	0.62	0.02	49.98	6.86	0.71	0.04
52	0.66	0.07	37.99	3.81	0.68	0.03
53	0.63	0.05	54.13	3.93	0.72	0.04
54	0.74	0.07	29.58	11.16	0.66	0.03
55	0.70	0.06	37.75	1.93	0.65	0.03
57	0.83	0.10	33.08	6.15	0.53	0.03
58	0.83	0.11	32.13	3.38	0.43	0.02
60	0.86	0.16	52.40	30.23	0.48	0.02
61	0.91	0.09	33.12	2.00	0.59	0.03
62	1.04	0.20	62.52	24.41	0.67	0.03
63	0.76	0.05	35.09	1.91	0.69	0.03
65	0.76	0.07	39.43	4.72	0.71	0.04
67	0.78	0.05	35.63	1.92	0.56	0.03
68	0.78	0.08	35.26	1.09	0.49	0.02
69	0.81	0.07	35.67	1.10	0.57	0.03
70	0.96	0.13	33.01	2.44	0.54	0.03
71	0.97	0.12	27.57	1.72	0.56	0.03
75	0.70	0.07	37.72	1.82	0.73	0.04
76	0.74	0.05	38.75	4.60	0.75	0.04
78	0.72	0.06	42.18	2.81	0.71	0.04
79	0.69	0.11	41.76	5.30	0.75	0.04
81	0.74	0.13	31.60	5.84	0.67	0.03

Table S6. Backbone chemical shift assignments (ppm) for free Δ10-ngMinE at 35ºC

Residue	$^{1}H_{N}$	^{15}N	$^{13}C\alpha$
A15	-	-	52.52
T16	8.14	114.18	61.93
V17	8.07	122.29	61.95
A18	8.36	128.22	52.47
R19	8.09	120.03	-
D20	8.29	120.93	54.64
R21	8.64	119.71	55.47
L22	8.74	120.80	53.71
Q23	9.34	125.81	54.69
I24	9.13	125.51	59.99
I25	9.50	128.07	59.89
I26	9.43	125.95	60.46
A27	9.38	128.77	50.13
Q28	9.14	122.57	54.69
E29	8.95	129.38	55.67
R30	8.43	124.03	54.51
A31	8.75	126.71	52.62
Q32	8.69	120.44	57.04
E33	8.63	119.17	57.18
G34	7.98	108.01	45.58
Q35	8.30	117.27	55.84
T36	7.99	118.08	60.70
P37	-	-	62.78
D38	7.89	116.80	53.20
Y39	9.68	125.92	60.01
L40	8.55	121.74	61.07
T42	7.30	113.92	66.12
L43	8.40	121.22	57.73
R44	8.89	119.86	60.51
K45	7.25	117.01	59.63
A46	7.92	120.22	54.68
L47	8.72	117.74	57.04
M48	8.00	117.55	58.39
E49	7.54	119.38	59.56
V50	7.93	118.68	66.07
L51	-	-	58.16
S52	7.88	112.94	61.34
K53	7.68	118.58	58.65
Y54	8.06	113.12	59.89
V55	7.80	117.07	61.44
N56	7.74	117.54	54.10
V57	7.33	114.72	60.11
S58	8.46	118.82	57.43
L59	8.54	123.13	57.85
D60	8.23	115.46	55.87
N61	7.94	116.42	53.63
I62	7.44	119.34	61.46
R63	8.64	127.53	55.27
I64	8.75	126.21	60.74

Table S6 (cont.)

Residue	$^{1}H_{N}$	^{15}N	$^{13}C\alpha$
S65	9.12	122.34	57.14
Q66	8.53	123.30	55.16
E67	8.87	123.67	55.05
K68	8.63	123.59	55.72
Q69	8.74	124.08	54.75
D70	9.03	124.32	56.16
G71	8.53	107.70	45.84
M72	7.96	119.51	54.86
D73	8.55	123.78	54.64
V74	8.65	121.53	61.67
L75	9.47	130.93	53.56
E76	9.19	126.05	54.73
L77	9.64	125.68	54.36
N78	9.04	121.50	53.57
I79	9.10	125.13	60.65
T80	8.62	124.38	62.55
L81	8.09	127.29	-
P82	-	-	62.97
E83	8.54	121.36	56.75
Q84	8.57	122.84	55.70

Table S7. Backbone chemical shift assignments (ppm) for bicelle-bound Δ10-ngMinE at 35°C

Residue	$^{1}H_{N}$	^{15}N	$^{13}C\alpha$
T14	8.13	115.71	61.78
A15	8.33	126.29	52.51
T16	8.13	114.19	61.92
V17	8.07	122.29	62.02
A18	8.35	128.22	52.47
R19	8.08	-	54.89
D20	8.30	120.97	54.65
R21	8.62	119.81	55.44
L22	8.77	120.91	53.68
Q23	9.32	125.78	54.67
I24	9.12	125.27	6xxxx
I25	9.53	128.04	59.86
I26	9.40	125.79	60.38
A27	9.39	128.71	50.11
Q28	9.12	122.35	54.66
E29	8.95	129.21	55.68
R30	8.42	124.03	54.53
A31	8.73	126.74	52.64
Q32	8.67	120.33	56.99
E33	8.62	119.27	57.17
G34	8.01	108.07	45.58
Q35	8.29	117.34	55.82
T36	8.00	118.05	60.67
P37	-	-	62.77
D38	7.91	117.00	53.29
Y39	9.62	125.70	59.86
L40	8.53	121.72	60.95
T42	7.31	113.92	66.21
L43	8.35	121.45	57.80
R44	8.88	119.60	60.49
K45	7.30	117.18	59.63
A46	7.94	120.32	54.75
L47	8.66	117.49	57.20
M48	8.03	117.33	58.40
E49	7.65	119.51	59.62
V50	7.92	118.42	66.07
L51	8.60	119.13	58.14
S52	7.90	112.62	61.29
K53	7.63	118.58	58.47
Y54	7.97	113.57	59.74
V55	7.82	117.01	61.50
N56	7.82	118.05	53.96
V57	7.43	115.53	60.35
S58	8.43	118.60	57.47
L59	8.53	123.06	57.65
D60	8.22	115.65	55.84
N61	7.95	116.29	53.66
I62	7.47	119.22	61.26
R63	8.63	127.24	55.24

Table S7 (cont.)

Residue	$^{1}H_{N}$	^{15}N	$^{13}C\alpha$
I64	8.75	125.98	60.59
S65	9.11	122.22	57.15
Q66	8.54	123.28	55.17
E67	8.86	123.62	55.07
K68	8.63	123.60	55.73
Q69	8.74	124.02	54.79
D70	9.01	124.29	56.17
G71	8.53	107.80	45.85
M72	7.96	119.58	54.75
D73	8.55	123.81	54.56
V74	8.65	121.47	61.63
L75	9.46	130.83	53.58
E76	9.19	125.99	54.71
L77	9.62	125.85	54.31
N78	9.04	121.76	53.49
I79	9.12	125.16	60.63
T80	8.61	124.12	62.51
L81	8.12	127.25	53.09
P82	-	-	62.97
E83	8.54	121.41	56.77
Q84	8.57	122.88	55.72

Table S8. Absolute differences in ^{15}N chemical shifts, $|\Delta\omega_{AB}|$, between the major and minor species of bicelle-bound ngMinE obtained from ^{15}N CPMG relaxation dispersion experiments.

Residue	$\|\Delta\omega_{AB}\|$ (ppm)
27	0.29 ± 0.17
29	0.65 ± 0.35
30	0.37 ± 0.20
31	0.34 ± 0.19
33	0.30 ± 0.17
34	0.28 ± 0.15
35	0.24 ± 0.13
36	0.28 ± 0.15
38	0.45 ± 0.24
40	0.37 ± 0.21
42	0.41 ± 0.22
52	0.73 ± 0.40
58	0.92 ± 0.51
61	0.38 ± 0.21
63	1.04 ± 0.59
67	0.45 ± 0.24
68	0.56 ± 0.30
69	0.45 ± 0.24
70	0.56 ± 0.30
71	0.87 ± 0.49
75	0.51 ± 0.27
78	0.95 ± 0.53

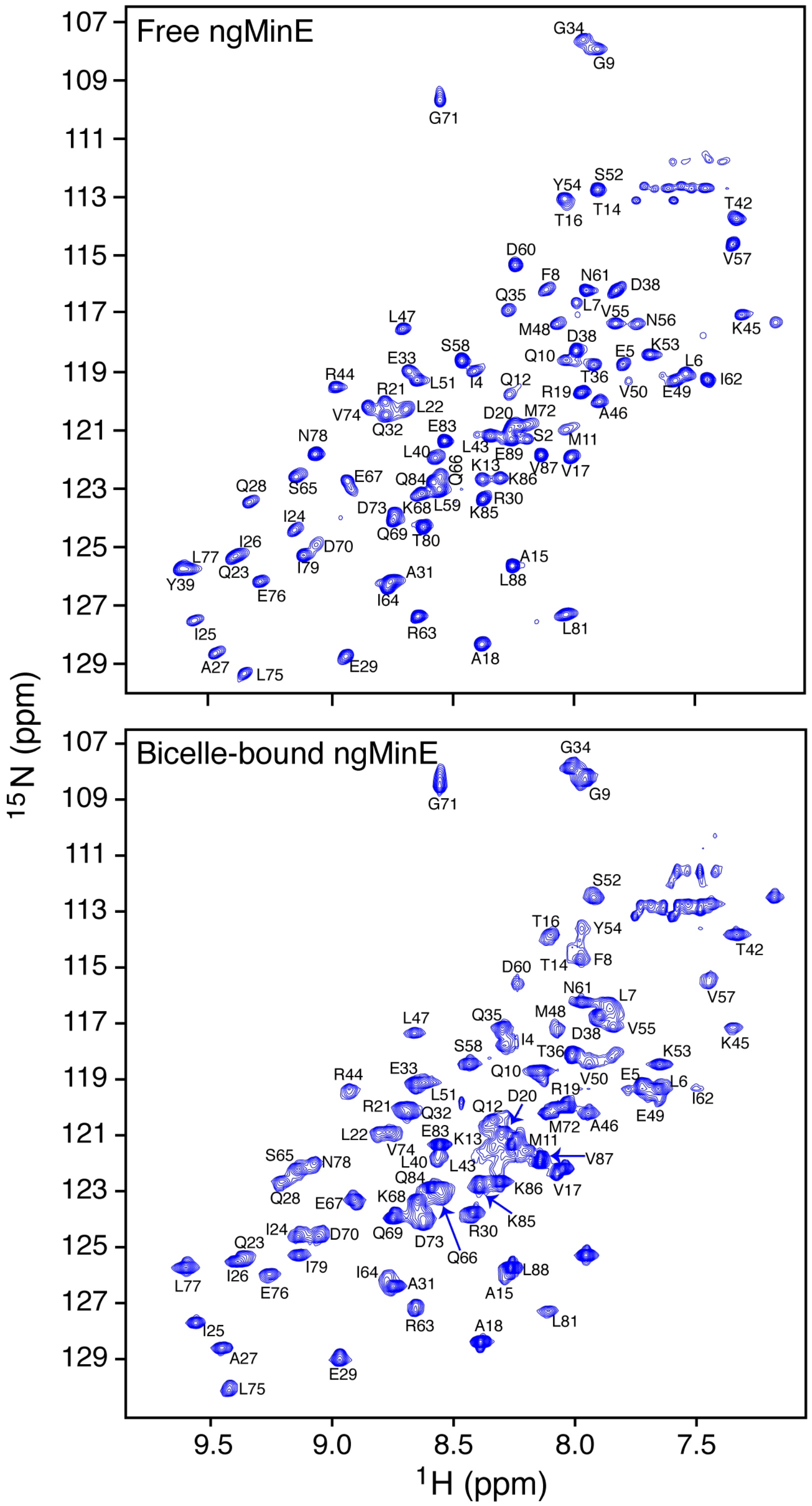

Figure S1. ^{1}H-^{15}N TROSY correlation spectra recorded on free and bicelle-bound ^{2}H/^{15}N-labeled ngMinE at 900 MHz and 35 °C. Experimental conditions: 0.5 mM (in subunits) ngMinE, 25 mM potassium phosphate, pH 6.5, 1 mM EDTA, 1 mM benzamidine chloride, 95% H_2O/ 5% D_2O (v/v), and, in the case of the bicelle-bound sample, 100 mM q = 0.5 DMPC:DHPC (comprising 33.3 mM DMPC and 66.67 mM DHPC). The latter corresponds to a bicelle concentration of ~1.2 mM with a molecular weight of ~ 70 kDa.

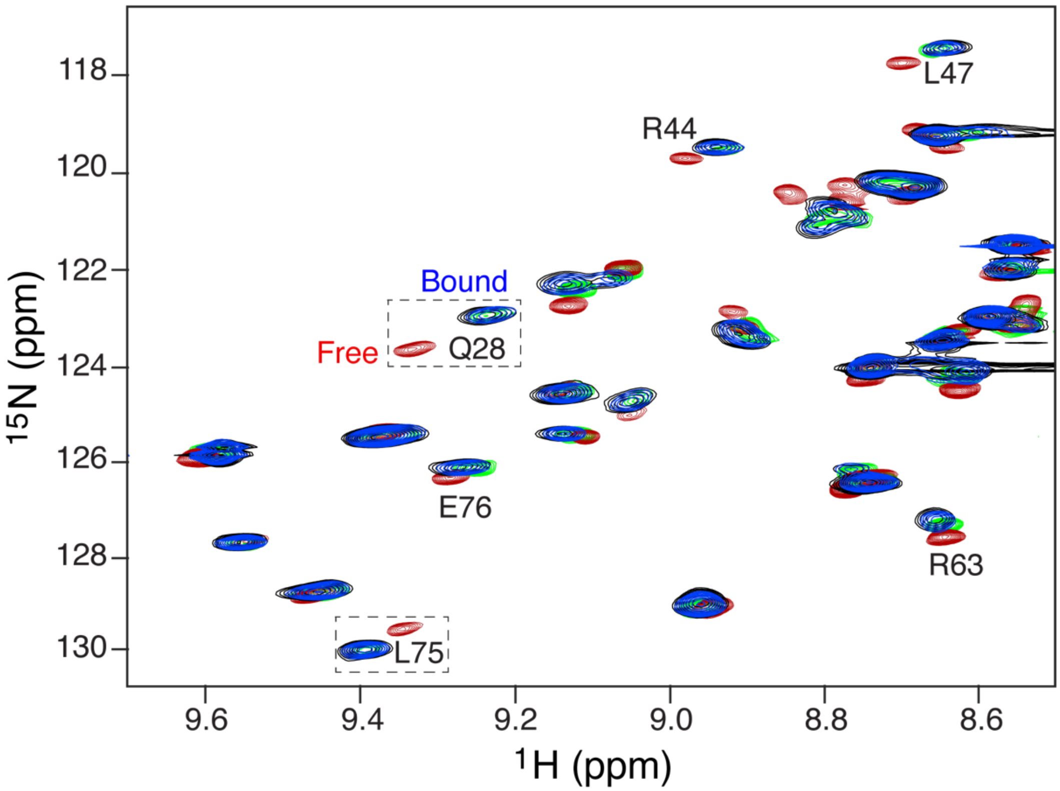

Figure S2. Portion of the ^{1}H-^{15}N TROSY correlation spectra of free and bound ^{2}H/^{15}N-labeled ngMinE. Red, 500 µM (in subunits) free ngMinE; green, blue and black, 50, 200 and 600 µM (in subunits) ngMinE, respectively in 60 mM q = 0.5 DMPC:DHPC bicelles (comprising 20 mM DMPC, 40 mM DHPC). Note that for some cross-peaks (e.g. L47, R63, E76), there are very small difference in shifts between the spectrum obtained with 50 µM ngMinE and those with 200 and 600 µM ngMinE in the presence of bicelles; this is due to small, unavoidable differences, in sample conditions (e.g. pH, exact bicelle concentration, etc…) and not to fast exchange between free and bound resonance positions since the small shifts for these three cross-peaks at different ngMinE concentrations in the presence of bicelles do not lie on a straight line trajectory relative to the corresponding free cross-peak (in red). All spectra were recorded at 600 MHz and 35°C in 25 mM potassium phosphate, pH 6.5, 1 mM EDTA, 1 mM benzamidine chloride, and 95% H_2O/5% D_2O (v/v).

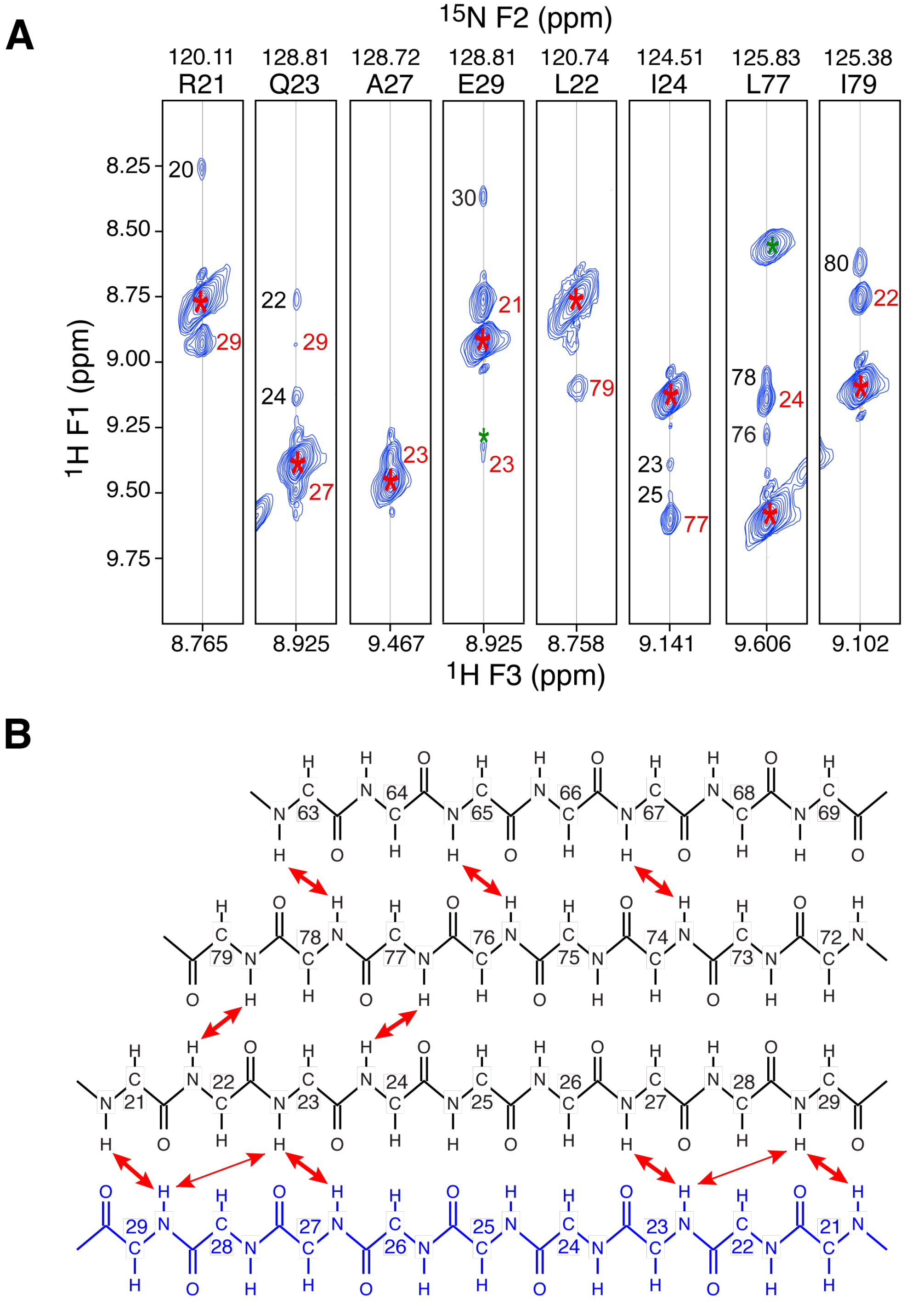

Figure S3. Backbone amide-backbone amide NOEs observed across the β-sheet region for bicelle-bound ngMinE. (A) Examples of strips ^{1}H(F1)-^{1}H(F3) strips from the 3D TROSY-based ^{15}N-separated NOE spectrum recorded at 900 MHz and 35 °C with a NOE mixing time of 150 ms. Interstrand cross-peaks are labeled in red, while sequential intrastrand cross-peaks are labeled in black. (B) Schematic of the β-sheet (strands β1 to β3 from one subunit in black, and the β1 strand from the second subunit in blue) with unambiguous interstrand NOEs shown by the arrows. The diagonal peaks are indicated by the red asterisks, and peaks whose intensities are maximal on a neighboring ^{15}N (F2) slice are indicated by the green asterisks. Thick and thin red lines indicate medium – strong and weak NOEs, respectively. The data were collected on ^{2}H/^{15}N-labeled ngMinE (0.5 mM in subunits) in 100 mM q = 0.5 DMPC:DHPC bicelles (comprising 33.3 mM DMPC and 66.67 mM DHPC).

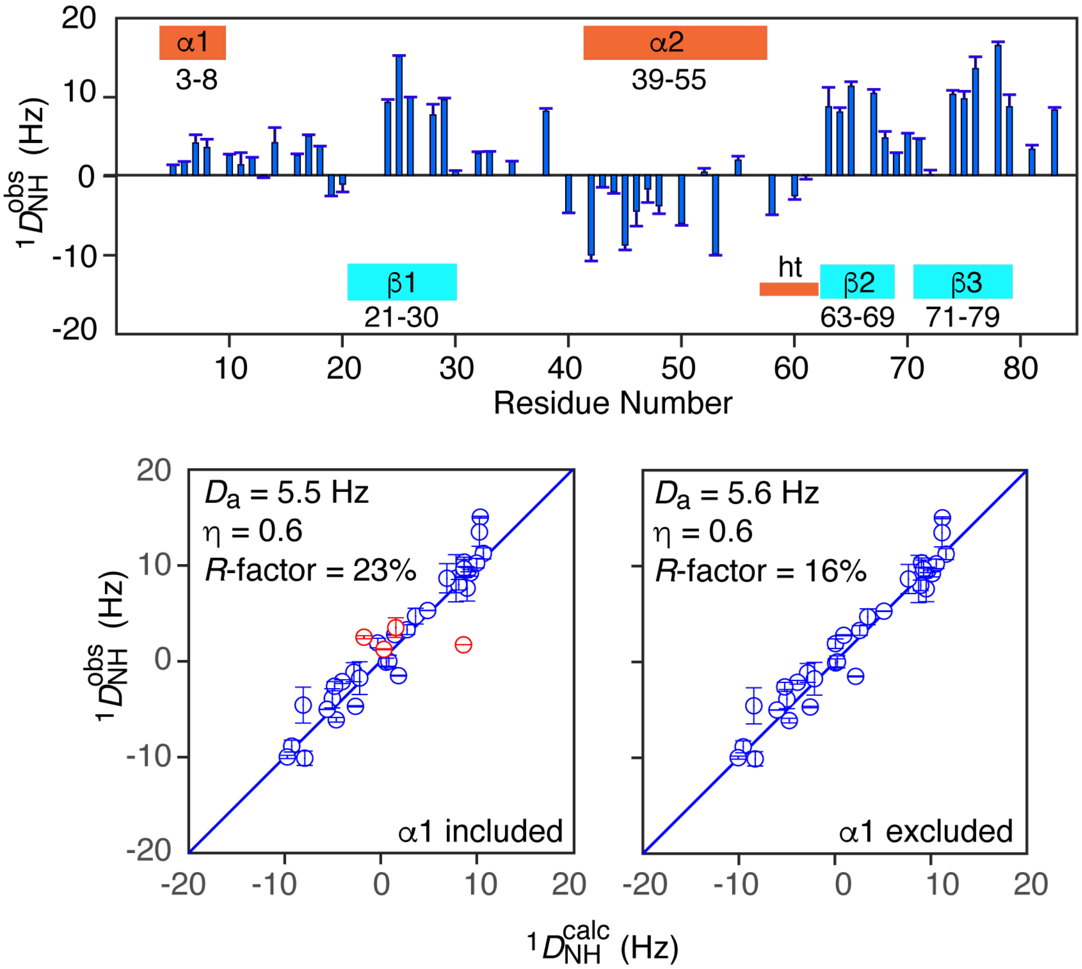

Figure S4. Backbone Amide RDC ($^1D_{NH}$) measurements on bicelle-bound ngMinE aligned in a positively charged stretched gel. (Top) Observed $^1D_{NH}$ profiles as a function of residue (see also Table S3); (Bottom) observed versus calculated RDCs including (left, red) and excluding (right) residues from helix α1. The calculated RDCs were based on the PBD coordinates (6U6P) of ngMinE (1). The RDC *R*-factors were calculated as $\{<(D_{obs} - D_{calc})^2>/(2<D_{obs}^2>)\}^{1/2}$ (2), and best-fitting was carried out using the CalcTensor helper function in Xplor-NIH (3). D_a and η are the magnitude of the principal component and rhombicity, respectively, of the alignment tensor. Only RDCs in elements of secondary structure are included in the correlation plots. The positively charged stretched gel comprises 4.16% (w/v) acrylamide, 0.11% (w/v) bisacrylamide, and 0.23% (w/v) 3-acrylamidoproly)-trimethylammonium chloride. RDCs were calculated as the difference in one-bond ^1H-^{15}N couplings measured in aligned (gel) and isotropic (water) media. The RDC data were collected on $^2H/^{15}N$-labeled ngMinE (0.5 mM in subunits) in the presence of 100 mM q = 0.5 DMPC:DHPC bicelles (comprising 33.3 mM DMPC and 66.67 mM DHPC) at 700 MHz and 35°C using the TROSY-based ARTSY method (4).

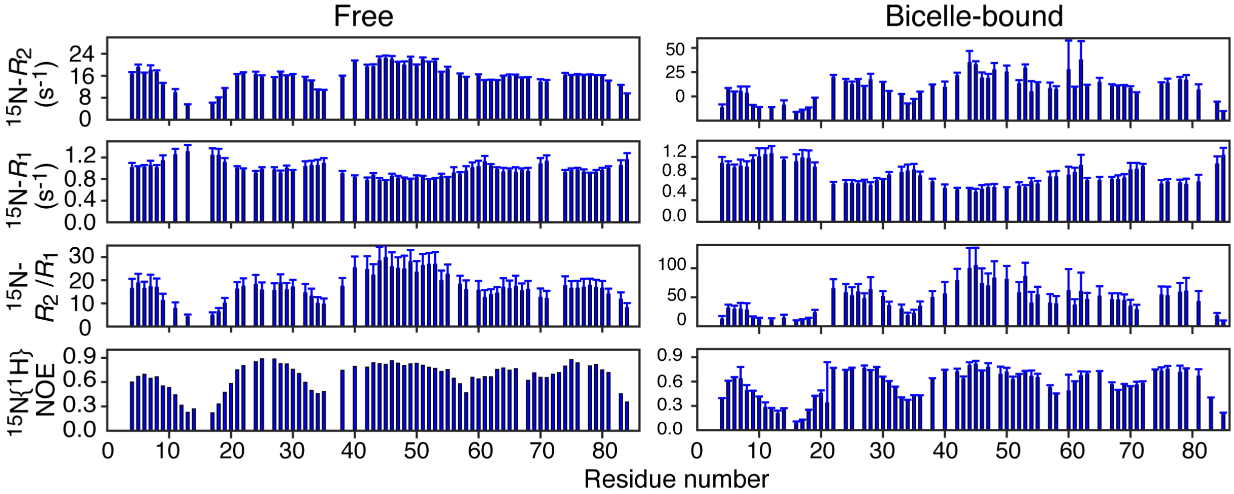

Figure S5. ^{15}N relaxation data profiles for free and bicelle-bound ngMinE measured at 700 MHz and 35°C. The concentration of ngMinE was 0.5 mM in subunits; the 100 mM q = 0.5 DMPC:DHPC bicelles comprise 33.3 mM DMPC and 66.67 mM DHPC.

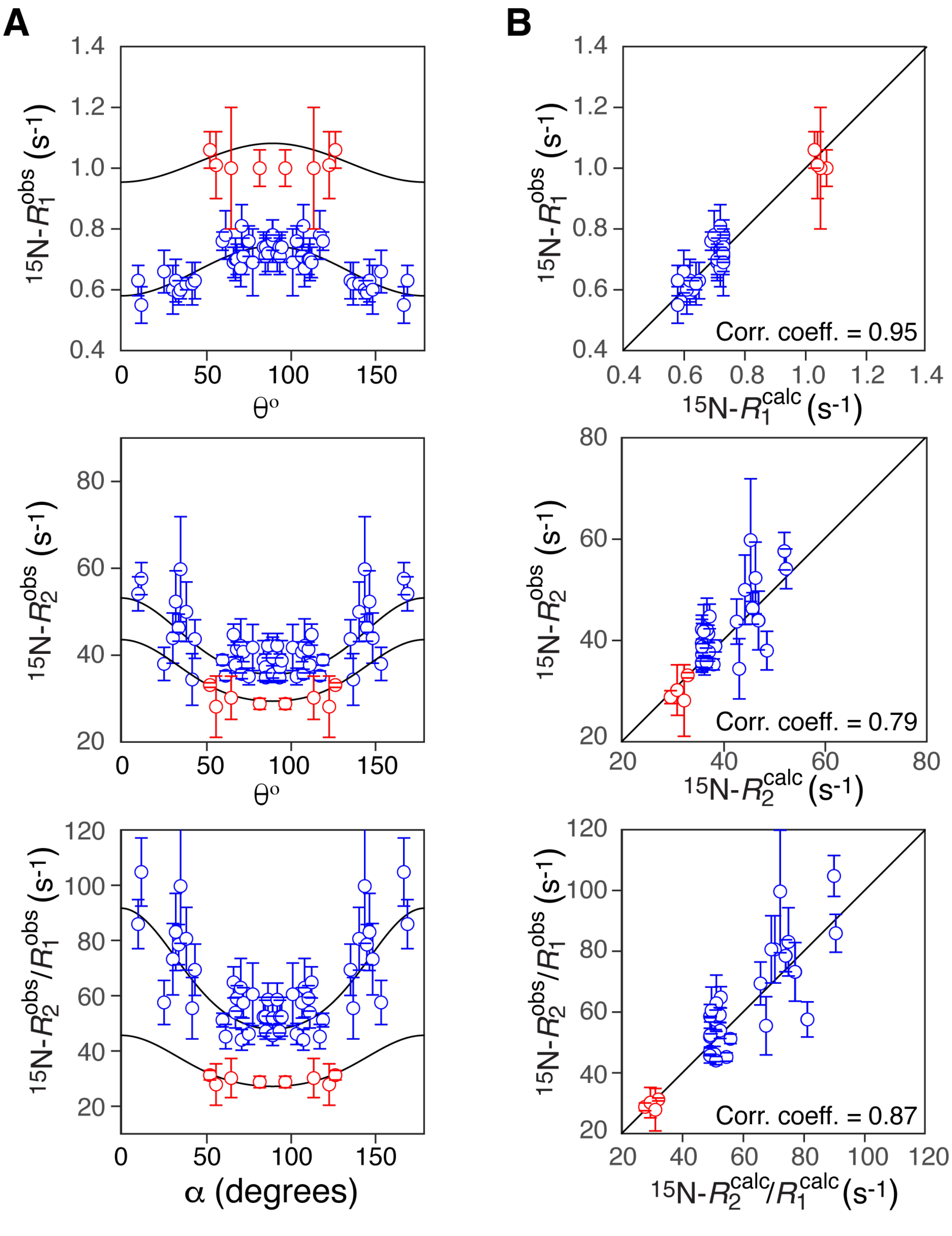

Figure S6. Fitting of ^{15}N relaxation data for bicelle-bound ngMinE using the coordinates of free ngMinE (PDB 6U6P) (1). (A) Plot of ^{15}N relaxation parameters (R_2, R_1 and R_2/R_1 ratio) versus the angle α that the N-H bond vectors subtend with respect to the principal axis of the diffusion tensor. The experimental data (see Figure S5) were recorded at 700 MHz and 35°C. The circles are the experimental data with the α1 helix residues in red and the remainder in blue (only residues in secondary structure elements were included in the fitting); the solid lines represent the best-fit curves obtained with the diffusion parameters given in Table 1 of the main text. (B) Correlation between observed and calculated ^{15}N relaxation parameters.

Supporting Information References

1. Cai, M., Huang, Y., Shen, Y., Li, M., Mizuuchi, M., Ghirlando, R., Mizuuchi, K., and Clore, G. M. (2019) Probing transient excited states of the bacterial cell division regulator MinE by relaxation dispersion NMR spectroscopy. *Proc Natl Acad Sci U S A* **116**, 25446-25455
2. Clore, G. M., and Garrett, D. S. (1999) *R*-factor, free *R*, and complete cross-validation for dipolar coupling refinement of NMR structures. *J. Am. Chem. Soc.* **121**, 9008-9012
3. Schwieters, C. D., Bermejo, G. A., and Clore, G. M. (2018) Xplor-NIH for molecular structure determination from NMR and other data sources. *Protein Sci* **27**, 26-40
4. Fitzkee, N. C., and Bax, A. (2010) Facile measurement of ^{1}H-^{15}N residual dipolar couplings in larger perdeuterated proteins. *J Biomol NMR* **48**, 65-70
